# Lipid-A-dependent and cholesterol-dependent dynamics properties of liposomes from gram-negative bacteria in ESKAPE

**DOI:** 10.1038/s41598-022-22886-7

**Published:** 2022-11-14

**Authors:** Juan Felipe Franco-Gonzalez, Alejandra Matamoros-Recio, Angel Torres-Mozas, Blanca Rodrigo-Lacave, Sonsoles Martin-Santamaria

**Affiliations:** Department of Structural and Chemical Biology, Centro de Investigaciones Biologicas Margarita Salas, CIB-CSIC, C/Ramiro de Maeztu, 9, 28040 Madrid, Spain

**Keywords:** Computational chemistry, Lipids

## Abstract

AntiMicrobial Resistance (AMR) is a worldwide health emergency. ESKAPE pathogens include the most relevant AMR bacterial families. In particular, Gram-negative bacteria stand out due to their cell envelope complexity which exhibits strong resistance to antimicrobials. A key element for AMR is the chemical structure of lipid A, modulating the physico-chemical properties of the membrane and permeability to antibiotics. Liposomes are used as models of bacterial membrane infective vesicles. In this work, coarse-grained molecular dynamics simulations were used to model liposomes from ESKAPE Gram-negative bacteria (*Escherichia coli, Klebsiella pneumoniae*, *Acinetobacter baumannii*, and *Pseudomonas aeruginosa*). We captured the role of lipid A, cardiolipin and cholesterol on liposome morphology and physico-chemical properties. Additionally, the reported antimicrobial peptides Cecropin B1, JB95, and PTCDA1-kf, were used to unveil their implications on membrane disruption. This study opens a promising starting point to understand molecular keys of bacterial membranes and to promote the discovery of new antimicrobials to overcome AMR.

## Introduction

AntiMicrobial Resistance (AMR), i.e. the ability of a microorganism (like bacteria, and viruses) to resist antimicrobial agents (such as antibiotics, and antivirals), has dramatically increased over the past three decades becoming a pandemic in the shadows^1^. Strains of particular concern are known by the acronym ESKAPE since they include Gram-positive bacteria *Enterococcus faecium*, and *Staphylococcus aureus*, and Gram-negative bacteria *Klebsiella pneumoniae*, *Acinetobacter baumannii*, *Pseudomonas aeruginosa*, and *Enterobacter species* (*e.g.*, *Escherichia coli* and *Salmonella minnesota*). In particular, the WHO antibiotic-resistant "priority pathogens" list intends to promote urgent research and development of new antibiotics^2^. The list highlights the particular threat of Gram-negative bacteria, since the complexity of the bacteria cell envelope provides higher resistance against antimicrobials than Gram-positive bacteria^1^.

The outer membrane (OM) of Gram-negative bacteria cell wall provides an extra layer of protection and acts as a selective barrier to most small drug molecules^1,3^ The mechanisms of resistance in Gram-negative bacteria often involve chemical alterations of lipopolysaccharides (LPS), a main component of the OM. LPS are in contact with the external environment of the bacteria and are essential for the bacterial survival and growth^1,3^. The LPS chemical structure consists of lipid A, core oligosaccharide and O-antigen (Figure S1), and it can be modified by host adaptation in order to enhance resistance against antimicrobial drugs, and to evade or reduce surveillance by immune receptors. Many of these chemical changes occur on the lipid A, which can adopt different acylation and phosphorylation patterns^4^. Another main lipid component of bacterial membranes are the phospholipids, with varying acyl chain length, saturation, branching, and charged head groups^5^. All these chemical changes in composition modify the membrane properties: fluidity, charge, permeability to antibiotics, and insertion and folding of outer membrane proteins^1,5^.

Most bacteria release membrane vesicles (MVs) that have important and diverse functions in the bacterial function and pathogenicity, such as secretion of proteins, nutrient acquisition, and interbacterial communication^6^. Outer membrane vesicles provide a mechanism of interaction with host cells during infection, whereas inner membrane (IM) vesicles and outer-inner membrane vesicles are involved in transport of virulence factors, DNA transfer, and interception of antibiotics^6^. Bacterial MVs incorporate specific host lipids and other molecules, such as cholesterol, and enter host cells via multiple routes including cholesterol-rich raft mediated endocytosis^5^. Studies of pathogenic bacterial vesicles have contributed to understand infection mechanisms and to promote drug discovery in this relevant field^1^. More specifically, the use of liposomes as biological membranes models has emerged as one of the main tools to study drug-membrane interactions and to explain AMR mechanisms^7^. Liposomes (artificial lipid vesicles) share several of the characteristics associated to MVs, such as their ability to carry water-soluble macromolecules in their lumen or hydrophobic proteins on their surface. These features make liposomes ideal models to mimic the specific properties of prokaryotic membranes and MVs, by introducing systematic changes in their lipid content and composition^7^. Computational studies of bacterial liposomes have emerged as a powerful tool to predict the activity of new drugs in modulating the properties and functionality of bacterial membranes^3^.

The spread of resistance mechanisms is a threat to global health that urgently requires research and development of new treatment alternatives^2^. In recent years, antimicrobial peptides (AMPs) have enjoyed a renaissance. AMPs are cationic agents produced by the host immune system as a defense mechanism for protection against many pathogens^8^. Usually, AMPs have a length of 18–20 amino acids and are rich in lysine and arginine residues^9^. Due to their cationic nature, they are attracted to the negatively charged bacterial OM, and disrupt the membrane via different mechanisms, such as channel formation and the so-called “carpet mechanism”, *i.e*., aggregation leading to disintegration of the bacterial cell wall^3^.

**Figure 1:**
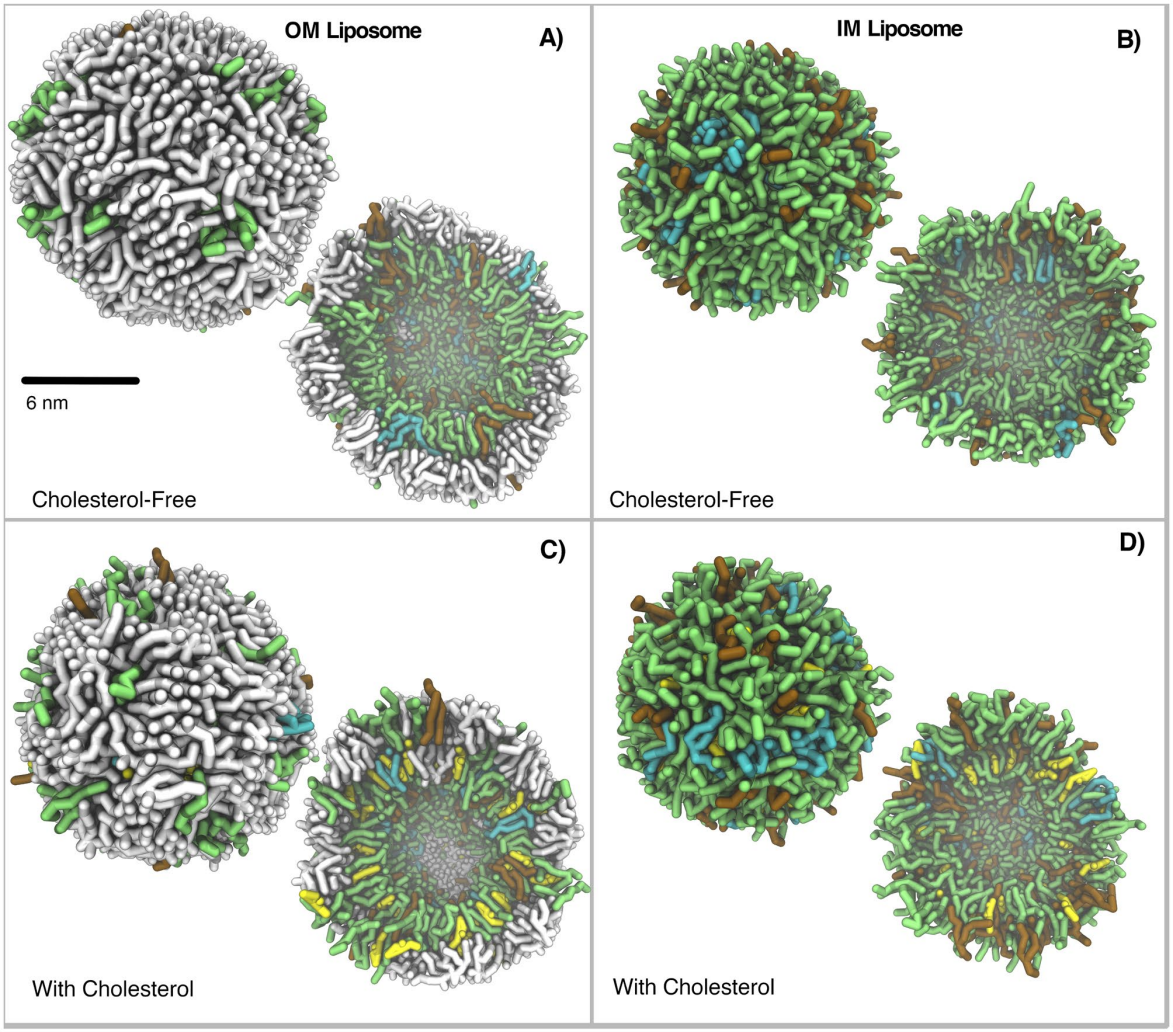
*E. coli* Liposome models. Cholesterol-free OM (**A**) and IM (**B**) liposomes respectively. With cholesterol (**C**) and (**D**) respectively. External view and a crossed-section view. Lipid A in white, Cholesterol in yellow, POPE in green, POPG in cyan and CDL in red. Water and Ca^+2^ beads are not included for clarification. 3D structures from last equilibrated frame.

In this work, heterogeneous lipid liposomes of four representative Gram-negative ESKAPE bacteria, *K. pneumoniae*, *A. baumannii, P. aeruginosa*, and *E. coli,* have been computationally modeled by means of coase-grained (CG) molecular dynamics (MD) simulations (Fig. 1). We have captured the role of lipid A and cholesterol on liposome morphology and physico-chemical properties. Moreover, three AMPs, including a polymyxin E, a polypeptide, and a plasticin, as representative antimicrobial agents, have been studied to investigate the implications of their reported anti-bacterial activity-related properties, such as membrane disruption and insertion into the lipid bilayer, on the structure and physico-chemical properties of bacterial liposomes. Our work opens promising avenues to understand the molecular factors determining the properties of the bacterial membrane and, therefore, provides new approaches for the study of AMPs to overcome bacterial resistance^1,3^.

## Results and discussion

The bacterial cell envelope is highly impermeable in AMR strains and it has attracted considerable attention as a target for novel antimicrobials effective against AMR. In particular, there is considerable interest in understanding the MV-mechanism for virulence factors transport, and for bacterial entry inside host cells^6^. In recent years, liposomes have attracted substantial attention as artificial models to analyze biological and physico-chemical properties in bacteria membranes and MVs^7^.

The transport of virulence factors through endocytosis has been shown to be mediated by lipid rafts, namely, plasma membrane domains rich in cholesterol content, which are present in a wide range of bacterial MVs. Moreover, it has been shown that pathogens incorporate cholesterol within the raft regions of host membrane to trigger their internalization into the host cells^5^. In particular, several studies reported interactions between ESKAPE bacteria and membrane rafts enriched by cholesterol during infection. For example, formation of cholesterol-rich membrane microdomains was reported in *A. baumannii* and *P. aeruginosa* MVs^10,11^. Furthermore, depletion of cholesterol (in vivo and in vitro), has been shown to restrict host-entry and prevent the invasion of various ESKAPE pathogens such as *E. coli*^12^, K*. pneumoniae*^13^, *A. baumannii*^10^, and *P. aeruginosa*^11^.

In this work, we have employed coarse-grained (CG) molecular dynamics (MD) simulations to understand the lipid A-dependent morphology and dynamics of both OM and IM liposomes in ESKAPE Gram-negative bacteria. We built OM and IM liposomes mimicking the lipid composition reported experimentally for strains of *K. pneumoniae*^14^, *A. baumannii*^15^, *P. aeruginosa*^14,15^, and *E. coli*^14^, summarized in Tables S1–4 (see “Methods” section). Furthermore, one additional liposome for each OM and IM liposome was used to simulate the impact of cholesterol on the liposomal morphology and dynamic properties. We note here that our OM liposomes include only the LPS lipid A moiety, rather than the full LPS structure. Main chemical modifications responsible for the membrane-targeting drug resistance of a given bacteria occur at the lipid A. Besides the additional computational cost of modelling full-length LPS structures, the outer polysaccharide is extremely variable among species and within the same species, and it is difficult to establish a common structure–activity correlation even for the same species. Conversely, lipid A is structurally more conserved among species^16^. Therefore, we decided to use lipid A as a minimal and representative LPS model to mimic the molecular and morphology features involved in bacterial OM vesicles.

To quantify the liposome morphology, structure, and dynamic properties, the following analyses were performed: area per lipid (APL), liposome size, ion stabilization, water permeation, and diffusion coefficient. We think that the library of representative ESKAPE Gram-negative bacteria liposomes can provide rational structure–property relationships between lipid A structural modifications and bacterial outer membrane properties. They can also help to uncover the role of acyl chain length (L_CHAIN_) and the number of acyl chains (N_CHAIN_) of the lipid A in regulating the physico-chemical properties of the membrane composition. In addition, our CG MD simulations were used to understand how cholesterol influences the bacteria membrane properties, specifically lipid ordering and membrane permeability.

## Liposome properties and cholesterol-incorporation effect

### Liposome morphology

**Figure 2:**
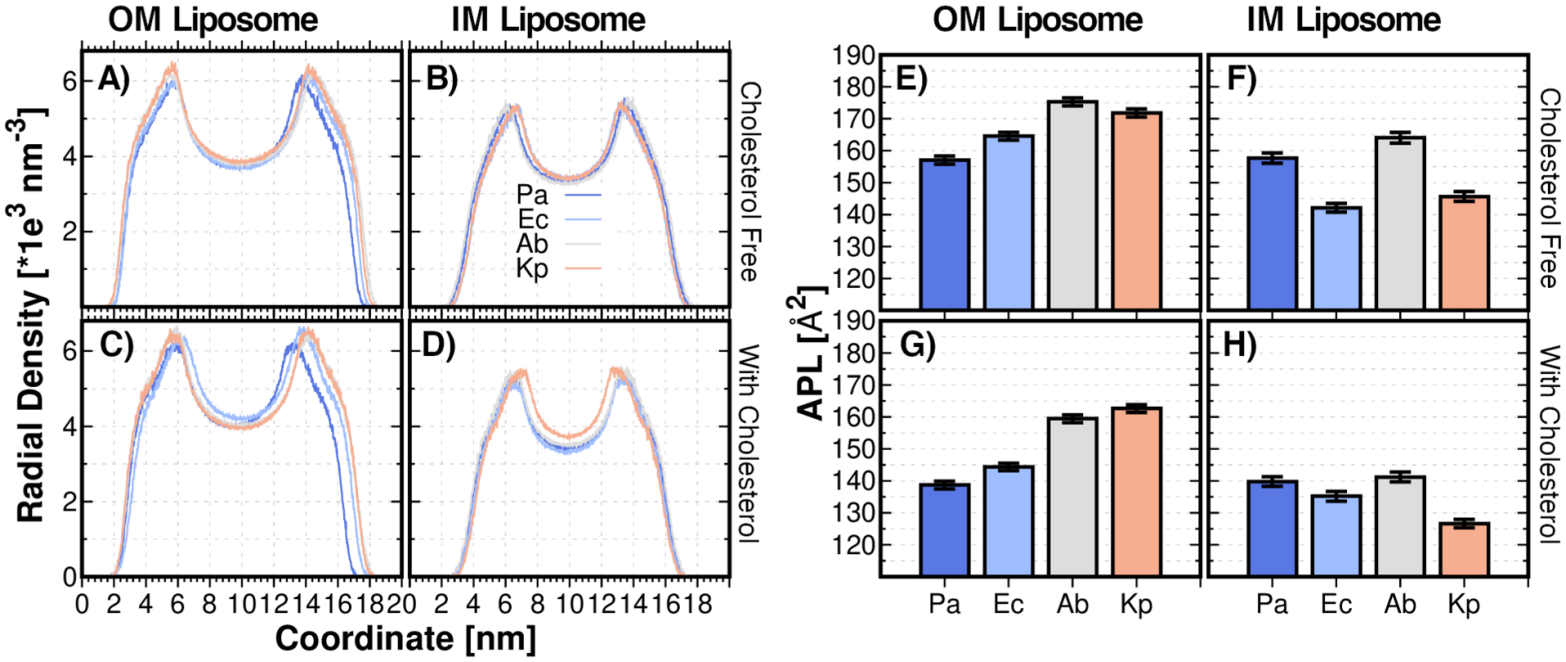
Liposome Components Density and APL. Liposome components density for, **(A**–**B)** both OM and IM liposomes respectively; **(C**–**D)** OM and IM liposomes containing cholesterol, respectively. APL measurements for, **(E**–**F)** both OM and IM liposomes, respectively; and, **(G**–**H)** OM and IM liposomes containing cholesterol, respectively. Kp: *K. pneumoniae*. Ab: *A. baumannii.* Pa: *P. aeruginosa*. Ec: *E. coli*. Analysis performed on the last μs of the MD production.

Liposome size can be estimated from the radial density of membrane components as shown in Fig. 2A–D. Our liposome models have diameters comprising values from ~ 14 to ~ 16 nm (Fig. 2A–D). Sizes are closer to the smallest membrane vesicles (MVs) known to be released by Gram-negative bacteria (10–400 nm in diameter)^17^. Therefore, our liposome models sizes are comparable to bacterial MVs in the in vivo scenario. Importantly, in the case of the OM vesicles, it is worth noting that these models do not include the antigen and core part of the LPS, the absence of which has been observed to produce a ≥ 10 nm decrease in the diameter of OM vesicles^17^.

The size of OM liposomes increases as the number of acyl chains of lipid A, N_CHAIN_, increases; the hepta-acylated lipids A of *K. pneumoniae* and *A. baumannii* lead to larger OM size values compared with the hexa-, and tetra-acylated lipid A molecules of *E. coli* and *P. aeruginosa*, *i.e.*, *K. pneumoniae* > *A. baumannii* > *E. coli* > *P. aeruginosa* (Fig. 2A, S2). As expected, when hepta-acylated lipid A molecules have the same *N*_CHAIN_, the OM liposomes size increases as the average C atoms per chain, *L*_CHAIN,_ increases (*K. pneumoniae* > *A. baumannii*), due to a larger number *N*_*CHAIN*_ and *L*_*CHAIN*_ implies a more voluminous lipid A molecule. Moreover, experimental and computational studies have shown the effect of the total number of acyl chains in lipid A on the stability of the OM; a larger *N*_*CHAIN*_ in lipid A increases the hydrophobic and van der Waals interactions between adjacent lipids, thus thickening the bilayer and leading to more tightly packed and ordered lipid tails^18^. The IM liposomes exhibit less marked variations in size than the OM liposomes, with the following trend: *A. baumannii ≈ P. aeruginosa* > *K. pneumoniae* ≈ *E. coli.* The slight increase in the IM size is a direct consequence of the greater presence of negatively charged phospholipids (*i.e.*, POPG or DOPG) and cardiolipin (Tables S1–4), which causes the increase in the liposomes leaflets cross-sectional area^19^.

As mentioned above, it is known that some bacteria, including ESKAPE species, incorporate cholesterol from host cells into liposomes during the bacterial colonization process^10–13^. Therefore, the cholesterol effect on liposome morphologies was also studied. Our simulations provide new information on cholesterol incorporation in the specific ESKAPE strains: *K. pneumoniae*, *A. baumannii, P. aeruginosa*, and *E. coli*. It is well-known that cholesterol incorporation induces changes in membrane lipid ordering and lipid packing^20^. We initially incorporated cholesterol in the inner leaflet of both OM and IM liposomes (Tables S1–4). After this starting location, cholesterol is free to spread in any direction. From the measurement of the liposome components density, it is observed that the liposomes feature changes in the size (Fig. 2C and D) with the incorporation of cholesterol. The membrane thickness (*L*) was estimated from the liposome density profile (Figure S7 and Table S5). Cholesterol insertion modified L in the cases of *E. coli* and *A. baumannii* OM liposomes, in which the membrane thickness increases with cholesterol incorporation. No relevant changes were observed in the cases of *K. pneumoniae* and *P. aeruginosa*. In the IM liposomes, only the membrane thickness of *E. coli* liposome decreases with cholesterol (Table S5).

The area per Lipid was also computed in the inner and outer surface of the liposome using a sphere with a radius of 4.5 Å as a probe. The calculated area was divided by the number of lipids present in the liposome to obtain an estimated APL. Figures 2E–H show the calculated APL for the OM and IM liposomes investigated in this work. A similar tendency as indicated in the radial density (*i.e*., liposome size) of membrane components is observed. *N*_CHAIN_ is correlated to the APL; the APL increases as *N*_CHAIN_ of lipid A increases; the hexa- and tetra-acylated lipids A of *E. coli* and *P. aeruginosa* have smaller APL compared with the hepta-acylated lipid A molecules (Figure S2). It seems that APL increases with *L*_*CHAIN*_ at a given *N*_*CHAIN*_. The fact the *A. baumannii* exhibits higher APL than *K. pneumoniae* is associated to the larger cardiolipin content in *A. baumannii*. It has been estimated experimentally, and by all-atom MD simulations, that APL/N_CHAIN_ increases as N_CHAIN_ decreases^4^. Interestingly, we also found the same relationships for APL/N_CHAIN_: 23.4, 21.9, 20.0, 19.6 and 20.9 Å^2^ for *P. aeruginosa, E. coli*, *A. baumannii,* and *K. pneumoniae*, respectively. This result shows that lipid A acyl chains are better packed with higher *N*_CHAIN_. Additionally, as it is expected, IM liposomes exhibit lower APL values than their counterparts do. The same trend for IM liposome size is observed for APL estimations. This is a direct consequence of a larger content of negatively charged lipids and, more specifically, of the presence of voluminous cardiolipin. APL increases as POPG, DOPG, and cardiolipin content increases (see liposome composition in Tables S1–4), thus, both *A. baumannii* and *P. aeruginosa* IM liposomes exhibit the largest APL values, and the rest of IM liposomes show the following relationship: *A. baumannii ≈ P. aeruginosa* > *K. pneumoniae* > *E. coli*.

The cholesterol-dependence of APL was also studied. The incorporation of cholesterol induces an ordered phase in lipid membranes which favors a compact lipid packing and, therefore, the APL decreases. In our simulations, the incorporation of cholesterol into OM and IM liposomes did indeed reduce the APL for all cases (see Fig. 2F,H). The APL/N_CHAIN_ value provides an approach to quantify the level of packing of lipid A acyl chains, and their ability to accommodate the rigid sterol ring^4^. Prior to cholesterol incorporation, the shorter chains of the tetra-acylated lipid A of *P. aeruginosa* featured low packing (APL/N_CHAIN_ = 23.4), possibly providing more space for the accommodation of cholesterol in the membrane and, consequently, exhibiting larger modifications of APL values than in the case of the other species (Fig. 2A–B, E–F).

### Ion stabilization

**Figure 3:**
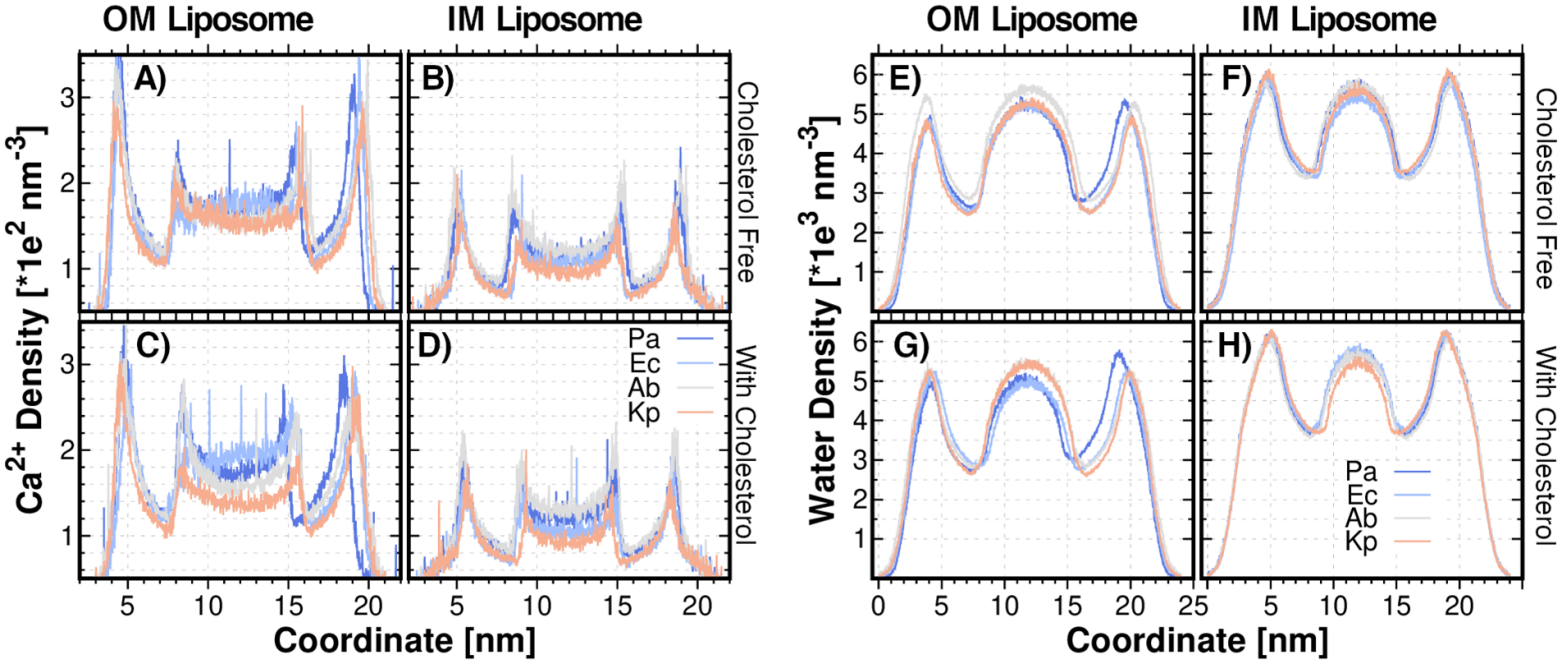
Radial density for ion Ca^2+^ and Water beads. Ca^2+^ number density in: **(A**–**B)** OM and IM liposomes, respectively; **(C**–**D)** OM and IM liposomes containing cholesterol, respectively. Water number density in: **(E**–**F)** OM and IM liposomes, respectively; and, **(G**–**H)** OM and IM liposomes containing cholesterol, respectivel. Kp: *K. pneumoniae*. Ab: *A. baumannii.* Pa: *P. aeruginosa*. Ec: *E. coli*. Analysis performed on the last μs of the MD production.

It is well-known that divalent cations, such as Mg^2+^ and Ca^2+^, play a stabilizing role in LPS-content liposomes^4^. It has also been shown that divalent cations stay on the lipid A membrane and stabilize the lipid A better than monovalent ions. Thus, our models included Ca^2+^ ions as the divalent cation of choice to neutralize the negatively charged lipid A and the rest of the lipid molecules. All the representative set of Gram-negative ESKAPE bacteria selected for this work have lipid A domains with two phosphate substituents (Figure S2). Consequently, the divalent cations distribution does not depend on the number of lipid A phosphate groups. The radial Ca^2+^ number density was calculated to understand the cation/lipid interaction. Density profiles are shown in Fig. 3A–D. It is observed that Ca^2+^ density decreases as lipid A N_CHAIN_ increases in OM liposomes, *i.e.*, *K. pneumoniae* < *A. baumannii* < *E. coli* < *P. aeruginosa* (Figure S2). Membranes with LPS of long N_CHAIN_ increase the hydrophobic and van der Waals interactions between adjacent lipids, thus strengthening the bilayer, while, in the case of LPS of shorter N_CHAIN_, with not sufficient hydrophobic and van der Waals interactions, the electrostatic repulsion is compensated in environments with high ionic strength where the excess of divalent cations cross-link the phosphate groups^18^. In our studies, as it was expected, the number density for Ca^2+^ ions in IM liposomes is lower than in OM liposomes due to the lipid A incorporation. In the IM liposomes, the Ca^2+^ number density is directly proportional to the presence of negatively charged phospholipids (*i.e.*, POPG and DOPG) and cardiolipin molecules (Tables S1–4). On this basis, it is evident that the number density profile is higher in *A. baumannii* IM liposomes due to a large number of Ca^2+^ ions incorporated to neutralize the cardiolipin and POPG content (15% and 30%, respectively, Table S4). Moreover, it has been reported that the permeability of phospholipid bilayers is greatly enhanced by the presence of cardiolipin^21^. Therefore, it becomes clear that the IM liposomes, with higher cardiolipin content, exhibit a higher Ca^2+^ permeability, and thus, a higher density in the hydrophobic core of liposomes (*A. baumanii* and *P. aeruginosa*, Fig. 3B and Tables S1, S4). Additionally, the incorporation of cholesterol does not exhibit a significant effect on the Ca^2+^ profiles for either of both IM and OM liposomes, except for *P. aeruginosa* OM liposome. This case is associated with the size reduction in the liposome caused by cholesterol, as shown in Fig. 2A,C. Nevertheless, cholesterol addition decreases the density of the Ca^2+^ ions around the outer leaflet of OM liposomes. This behavior becomes evident, since cholesterol incorporation into the lipid membrane stabilizes the membrane, as it reduces, on the one hand, the repulsion of charged headgroups by increasing the headgroup spacing and, on the other hand, the motion of hydrocarbon chains by increasing van der Waals interactions^20^. Thus, a lower density of cations is needed to minimize the electrostatic repulsions among adjacent lipid A and lipid molecules. Additionally, in the hydrophobic core region of OM liposomes, a pronounced curvature for the Ca^2+^ profile is observed after the cholesterol incorporation. It is related to the ion permeability of the membrane. It is widely accepted that the presence of cholesterol in membranes decreases membrane fluidity and, consequently, the membrane permeability for ions and small molecules decreases as well^20^. For these reasons, one might consider that the Ca^2+^ density in the hydrophobic core of the liposomes, *i.e.*, in the internal aqueous cavity, should decrease after cholesterol incorporation. Nevertheless, the usual assumption that permeability is controlled by fluidity should be regarded with caution in the case of ions. One notable exception is the case of liposomes containing mixed phospholipids, prepared in the presence of CaCl_2_. According to reported experimental data, the increment of the CaCl_2_ concentration leads to the reduction of the fluidity with essentially no effect on the ion permeability^22^. These results can give a reasonable explanation for our observations. However, additional work is needed to get quantitative data on this point.

### Hydration water

Unilamellar vesicles (liposomes) are susceptible to water permeation. In order to study the effect of lipid A and cholesterol on water permeation and water intake, the water number density was calculated as shown in Figs. 3E–H. In the OM liposomes, the hydration interface directly correlates with the APL parameter; *A. baumannii* > *K. pneumoniae* > *E. coli* ≈ *P. aeruginosa*. Larger lipid areas require more water at the membrane interfaces; the greater APL, the hydration interface increases. Regarding the water intake, the lower N_CHAIN_ in lipid A, the stronger the membrane permeability^23^. This agrees with the water density in the inner cavity of the OM, as observed in Fig. 2E (*K. pneumoniae* > *A. baumannii* ≈ *E. coli* ≈ *P. aeruginosa*). Moreover, in both OM and IM liposomes, the water density inside the inner cavity directly correlates with the inner cavity volume (Figs. 2A,B and 3E,F). Due to the size difference of the liposomes, it is expected a bigger amount of water in the inner region of the OM liposomes than in IM liposomes. This is because lipid A-content liposomes assemble a bigger hydrophobic core. Additionally, it seems that the hydration interface increases with a higher content in cardiolipin (Fig. 3F). Furthermore, a subtle increment in water density in the water/lipid interface is exhibited in both IM and OM liposomes when cholesterol is incorporated (Fig. 3E–H). The most evident change occurs in the case of the *P. aeruginosa* OM liposome. In this case, the modification on the water density distribution profile is a direct effect of the reduction in the liposome size caused by cholesterol, as shown in Fig. 2A–D. This effect was also reported in all-atom MD simulations of phospholipid bilayers, where it was observed an increase of the average distance between phospholipid head groups in the presence of cholesterol, and a tendency of water molecules to fill the voids^20^.

### Diffusion coefficient

**Figure 4:**
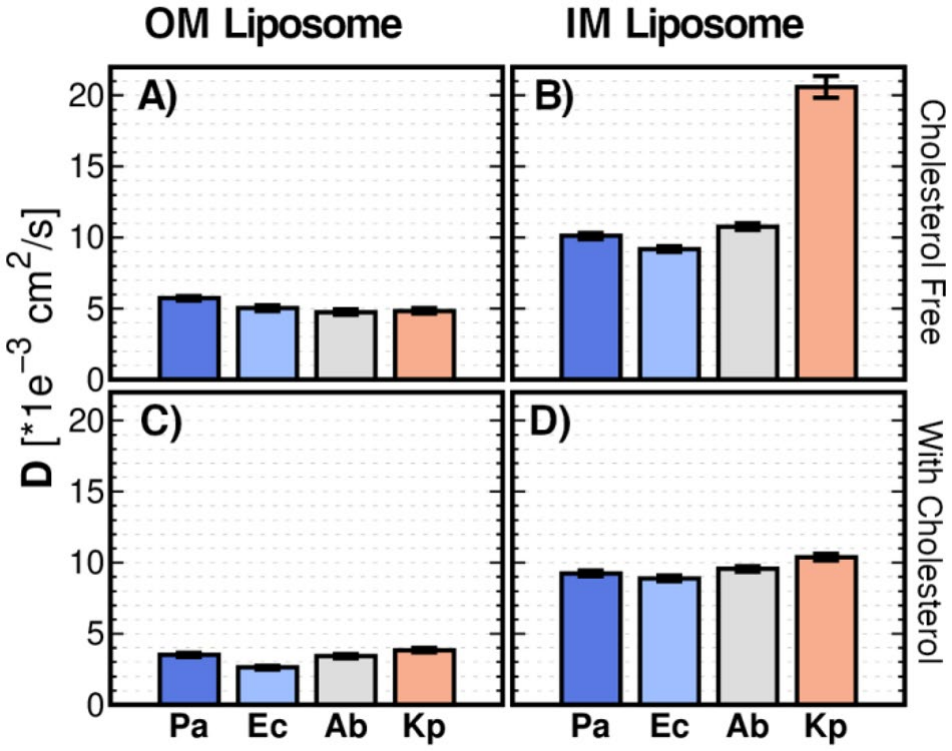
Diffusion Coefficient (D). Mean Diffusion coefficient from each individual molecule in: **(A**–**B)** OM and IM liposomes, respectively; **(C**–**D)** OM and IM liposomes containing cholesterol, respectively. Analysis performed on the last μs of the MD production.

The mean lateral diffusion coefficient (〈D〉) in the liposome membrane was calculated from the radial mean square displacement (MSD) on each individual molecule. In this study, the diffusion was fitted from the lineal region in the last μs of the production run. Figure 4 shows that lipid A incorporation reduces the diffusion in OM liposome membrane when compared with IM liposomes, which leads to a reduction of membrane fluidity. It can also be observed that 〈D〉 decreases as lipid A *N*_*CHAIN*_ increases (Fig. 4A). It agrees with previous all-atom simulations^4^. The slow diffusing bulkier hepta-acylated LPS restricts the diffusion of membrane lipids, more than the hexa- and tetra-acylated LPS. Note that our liposome model consists of heterogeneous mixtures of lipids. Therefore, changes in 〈D〉 are related to the features involved in domains formation (Figure S8)^24^. Although heterogenous lipid mixtures can be hard to study, our simulations reveal a clear pattern. *P. aeruginosa* and *A. baumannii* show a similar 〈D〉 value which is associated with their equivalent cardiolipin content. Cardiolipin, with its repulsive charged headgroups exhibits a larger diffusion coefficient than the zwitterionic counterparts PC lipids which is in agreement with what can be expected from headgroup areas^24^. Additionally, *K. pneumoniae* IM liposome, as an exceptional case, shows a two-fold increment of 〈D〉. It can be explained by the presence of *dioleoyl-* (DO) fatty acids DOPE and DOPG. It seems that pure DOPE systems have a greater 〈D〉 than POPE and, by extension, DOPG greater than POPG^25^.

Figure 4C,D show 〈D〉 values when cholesterol is incorporated into the liposome membrane. In both OM and IM liposomes 〈D〉 decreased. Again, in agreement with what is reported experimentally in literature^24^. A possible explanation for heterogenous systems such as the ones here presented, could be that the large size of the domains of lipids formed will mainly move within the same domain during the time of measurement (1 μs) and, therefore, exchange between the phases will be slow on this timescale.

## Liposome-antimicrobial peptide interaction

It is well-known that bacterial membrane morphology can adapt to the action of lipophilic agents such as antimicrobial peptides (AMP)^1^. Peptides and lipids are highly dynamic. Consistently, the peptides and lipids can form a wide variety of supramolecular assemblies^3^. In general terms, hydrophobic sequences preferentially adopt transmembrane alignments and form oligomeric structures similar to transmembrane helical bundles. On the contrary, charged amphipathic sequences tend to intercalate at the membrane interface and induce pronounced disruptions in the lipid conformation and packing^8^. As a result, the formation of membrane pores and disruption occur^3^. Among the adapting modifications by lipid bilayers to peptides action, there is an increased structuring of lipid bilayer^1^. The level of AMP binding and insertion has been shown to depend on the lipid composition and, therefore, on the physico-chemical properties of the membrane bilayers.

**Figure 5:**
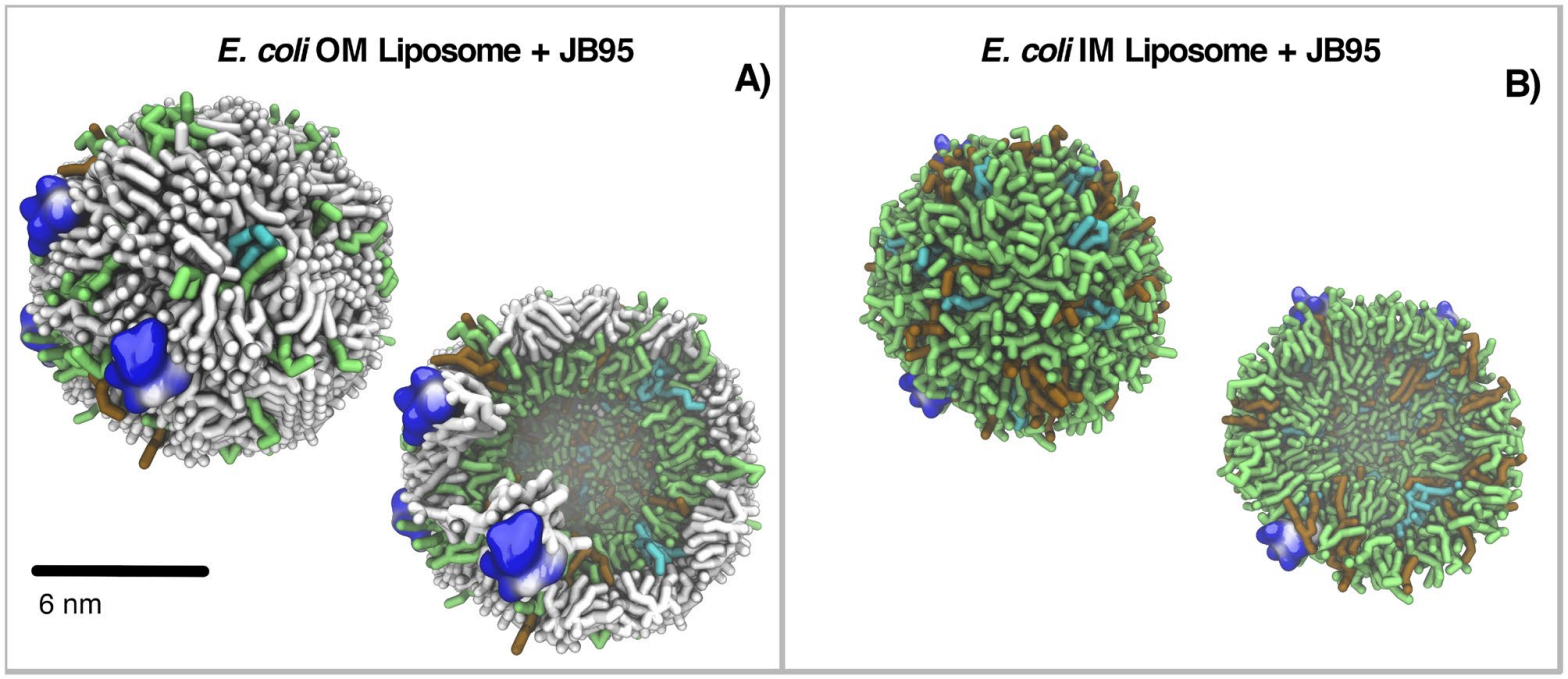
JB95-*E. coli* liposome interaction. (**A**) JB95-OM liposome and (**B**) JB95-IM liposome for both external and crossed-section views. Lipids are represented in sticks where lipid A is in white, POPE in green, POPG in cyan and CDL in red. JB95 is render by volumetric potential surface where blue represents postive charged aminoacids and white represents hidrophfocbic aminoacids. Water and Ca^+2^ beads are not included for clarification. 3D structures from last equilibrated frame.

The mechanism of action of membrane-targeting AMPs comprises two stages: (i) membrane binding, and (ii) peptide insertion into the hydrophobic core of the membrane^26^. Having established the role of lipid A and cholesterol in liposomes morphology, we next simulated the AMP-liposome interaction (*i.e*., first stage), to determine the AMP effect on liposome morphology and their physico-chemical properties. It is very well-known that AMPs have great potential as antimicrobial agents. Nevertheless, this potential has not yet resulted in an FDA-approved drug. Therefore, extensive structure–activity relationship studies need to be conducted to improve the physico-chemical properties of AMPs. Taking this into account, we chose AMPs with Minimum Inhibitory Concentration (MIC) values reported in the literature for any of the strains studied in this work^27–30^. We found the following AMPs with low MIC (MIC ≤ 1 μM) values studied in three Gram-negative ESKAPE strains (*P. aeruginosa*, *E. coli,* and *K. pneumoniae)*: a polymyxin E, JB95^27^; a polypeptide, Cecropin B1^28^; and, a plasticin PTCDA1-kf^31^ (Figure S3 and Table S6, and MIC values in Table S7). On this basis, the simulations were performed using the peptides JB95, Cecropin B1, and PTCDA1-kf against OM and IM liposomes of *E. coli*, *P. aeruginosa,* and *K. pneumoniae*. First, peptides were submitted to all-atom MD simulations in water, in order to reach a stable secondary structure (Figure S5 and Table S4). Then, liposomes were surrounded by four AMPs molecules, initially located randomly in the (external) aqueous phase (Fig. 5) by means of MARTINI CG MD simulations.

Cationic AMPs typically exhibit a balance between positively charged and hydrophobic amino acid residues that allow them to adopt an amphipathic conformation^8^. Due to the short sequences of most AMPs, their structures are highly dependent on the environment. Some AMPs are unstructured in aqueous solvent and undergo conformational changes in the hydrophobic membrane medium. They adopt a secondary structure such as α-helix, like AMPs Cecropin B1 and PTCDA1-kf, whereas others adopt β-sheets structures, as reported for JB95 (Figure S4 and Table S5)^32^. The membrane surface binding of polycationic peptides is mainly driven by electrostatic interactions between the peptides and the anionic microbial surface. The amphipathic conformation maximizes both, electrostatic and hydrophobic interactions with the membrane as the positive face promotes binding to the anionic headgroups, whereas the nonpolar face favors contact with the hydrophobic part of the membrane and allows insertion of the molecule into the hydrophobic core of the lipid bilayer^1^. In contrast to bacterial membranes, the outer monolayers of eukaryotic membranes are composed of zwitterionic (overall neutral) lipids, thereby partly explaining selectivity between eukaryotic and prokaryotic cells^32^. Importantly, MARTINI force-field restrains protein structure to the input conformation without reproducing folding dynamics. Since peptides conformations were obtained from atomistic simulations in aqueous medium, it is important to note that here we investigate the binding of the peptides to liposomes, *i.e*., the first stage involved in the mechanism of most membrane-targeting AMPs.

### AMP-liposome binding

**Figure 6:**
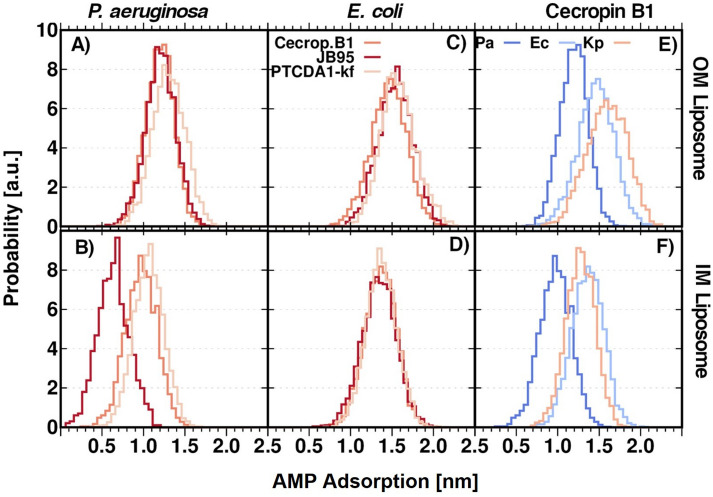
AMP Interfacial adsorption depth on: (**A**–**B**) *P. aeruginosa* OM and IM liposomes respectively; (**C**–**D**) *E. coli* OM and IM liposomes respectively. (**E**–**F**) Cecropin B1 adsorption depth of various OM and IM liposomes. Analysis performed on the last μs of the MD production. [*Image: “adsorption” corrected].

During MD simulations, AMPs binding to the liposome surface was observed (Fig. 5). Interfacial adsorption of AMP into the liposome bilayer was measured^33^ and it is shown in Fig. 6. It can be observed that an adsorption depth up to 1.5 nm from membrane surface can be reached by the studied AMPs. Generally, AMPs achieve a deeper interfacial adsorption in OM liposomes that their corresponding IM liposomes. The presence of negatively charged lipid A molecules increases the negative net charge of the OM surface. Consequently, a higher negative surface membrane potential in the OM significantly enhances the electrostatic interaction between the cationic peptides and the membrane^1^. Moreover, *E. coli* liposomes are more susceptible to AMP penetration that *P. aeruginosa* liposomes. This behavior is different to the one that could be expected a priori, since cationic peptides prefer an anionic membrane instead of a zwitterionic membrane^29^. Indeed, *P. aeruginosa* liposomes contain a higher amount of negatively charged phospholipids than *E. coli*, in both OM and IM (Tables S1 and S2). Nevertheless, since cardiolipin content is higher in *P. aeruginosa* than in *E. coli* (11 and 5% for *P. aeruginosa* and *E. coli* IM, respectively, Tables S1 and S2) our results are consistent with experimental observations where membranes containing cardiolipins are less sensitive to the AMP effect than those membranes containing another type of anionic lipids^34^. Besides the anionic charge, it has been suggested that the quadruple-chain structure of cardiolipin leads to a high degree of cohesion in the interfacial region of a cardiolipin containing bilayer and results in an increase in the structural integrity of the bilayer^35^. The relationship between the cardiolipin proportion and the AMP interfacial adsorption depth is clearly reflected in the Fig. 6E,F. As observed, Cecropin B1 is deeper adsorbed in the *P.* aeruginosa OM and IM liposomes and penetrates deeper and at a similar depth in *E. coli* and *K. pneumoniae* OM liposomes, which have almost the same cardiolipin content (5 and 6%, respectively). In OM liposomes, Cecropin B1 adsorption correlates with the lipid A N_CHAIN_, increasing the AMP adsorption (deeper adsorbed) as the N_CHAIN_ increases^23^. Interestingly, the AMP adsorption depth also agrees with Lethal Concentration (LC) reported for these AMPs: the lower is LC, the deeper is AMP adsorption.

### APL

**Figure 7:**
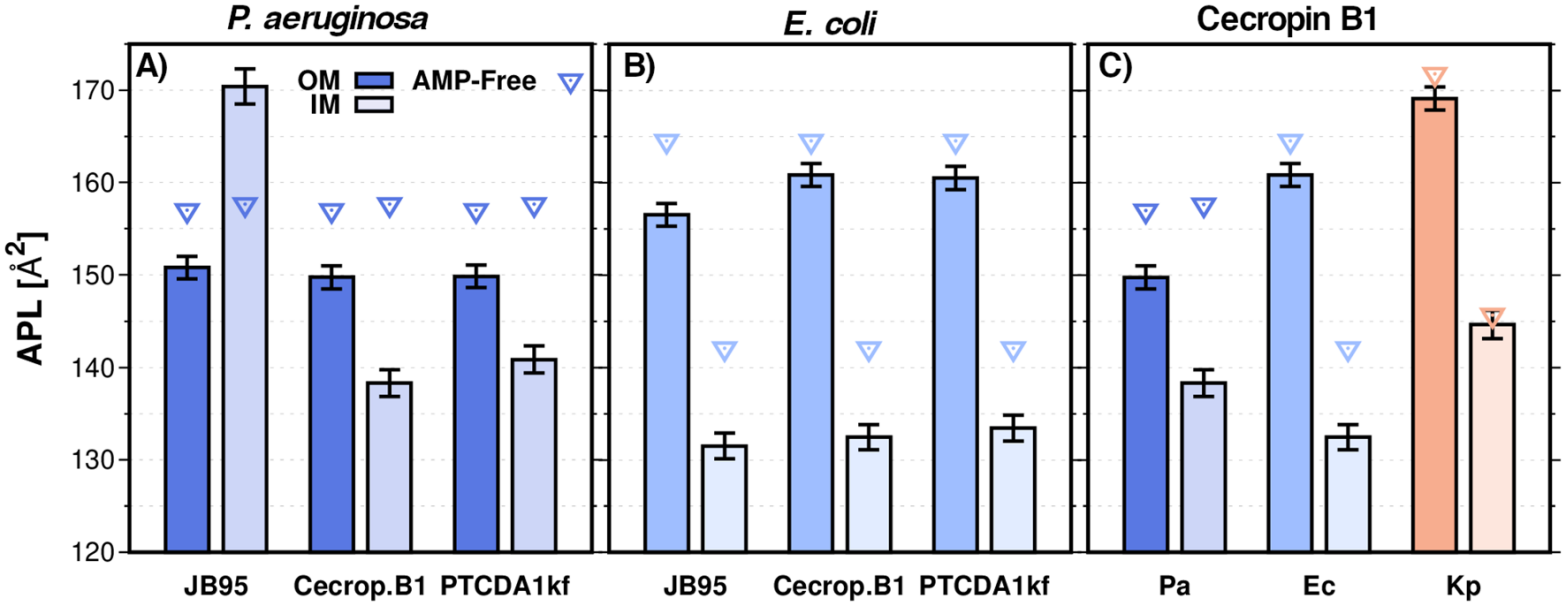
AMP-Dependent APL. (**A**–**B**) P. aeruginosa and E. coli liposomes. (**C)** Cecropin B1 effect on APL of some bacterial liposomes. Filled boxes correspond to OM liposomes and semi-transparent boxes correspond to IM liposomes. Analysis performed on the last μs of the MD production.

**Figure 8:**
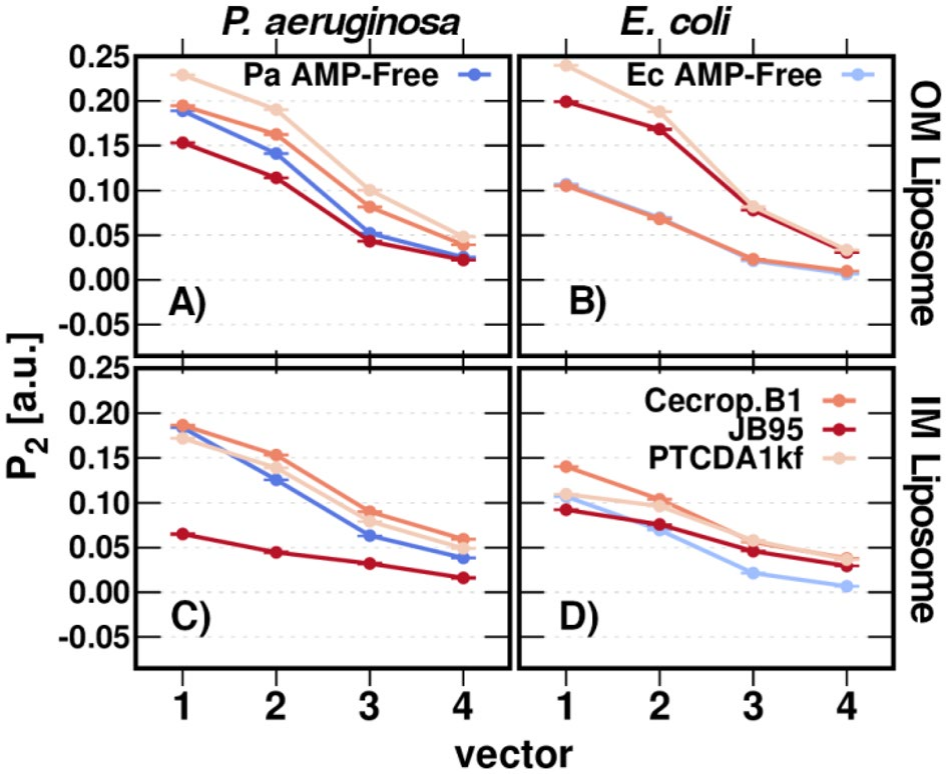
Order Parameter, P_2_. (**A**–**B**) *P. aeruginosa* and *E. coli* OM liposomes, respectively; (**C**–**D**) *P. aeruginosa* and *E. coli* IM liposomes, respectively. Analysis performed on the last μs of the MD production.

Figure 7 shows the APL estimations for the IM and OM liposomes models when the AMP is present. Except for *P.aeruginosa* IM liposomes in the presence of JB95, in all cases, APL values decreased with the presence of AMP. This is a consequence of the bacterial membrane adaptation to the AMP adsorption to the aqueous-bilayer interface; the incorporation of the AMPs induces a more ordered phase in lipid membranes and, therefore, APL is reduced. The increment in the ordering of the lipid molecules caused by the peptide agents is balanced by changing the configuration of the aliphatic chains of the unsaturated phospholipids from a modeled *cis* to a *pseudo cis*. This is not surprising, since phospholipids containing trans-unsaturated fatty acids show a reduced APL when compared with those containing corresponding *cis*-unsaturated fatty acids, indicating an increase in van der Waals attraction^36^. The order parameter, P_2_, was calculated for each individual fatty chain from all phospholipids and plotted as an average for each chain component in Fig. 8. It can be observed that, in general, P_2_ increases for most of the aliphatic chain components when the liposome interacts with a given AMP. It correlates with the variation in APL estimations, which are lower as lipid packing improves (P_2_ increases). The parameter P_2_ also reveals that JB95 induces a lipid disorder in *P. aeruginosa* IM liposomes and then increases APL, although JB95 does not penetrate the *P. aeruginosa* IM. However, JB95 still shows a low MIC (Table S7). According to our simulations, since JB95 does not penetrate the inner bacterial membrane (Fig. 6B), this polymyxin remains on the membrane surface and then, it generates a lipid package disorder which involves an increase of APL value and P_2_ reduction. Remarkably, for some specific AMPs, no membrane insertion has been observed experimentally, despite clear experimental evidence of membrane leakage and antibacterial activity^9^. Instead, the AMPs are adsorbed on the bacterial membrane and form hydrogen bonds with the lipid phosphate groups, disrupting the salt-bridges between phosphate groups and divalent cations, and destabilizing the close packing of the membrane^1,37^.

The AMPs interfacial adsorption in OM and IM correlates with the APL measurements Δ(APL_*AMP.free*_ − APL _*w.AMP*_). This means a deeper AMP adsorption lower Δ(APL_*AMP.free*_ − APL _*w.AMP*_). It is attributed to the steric hindrance caused by the presence of the AMPs in the bilayer. Indeed, our data show a direct relation between the peptides deep of adsorption and the perturbation in the membranes, which is responsible for more fluctuations in the lipid acyl chains, thus preventing the cis–trans conversion of unsaturated phospholipids.

### Water permeation and ion stabilization

**Figure 9:**
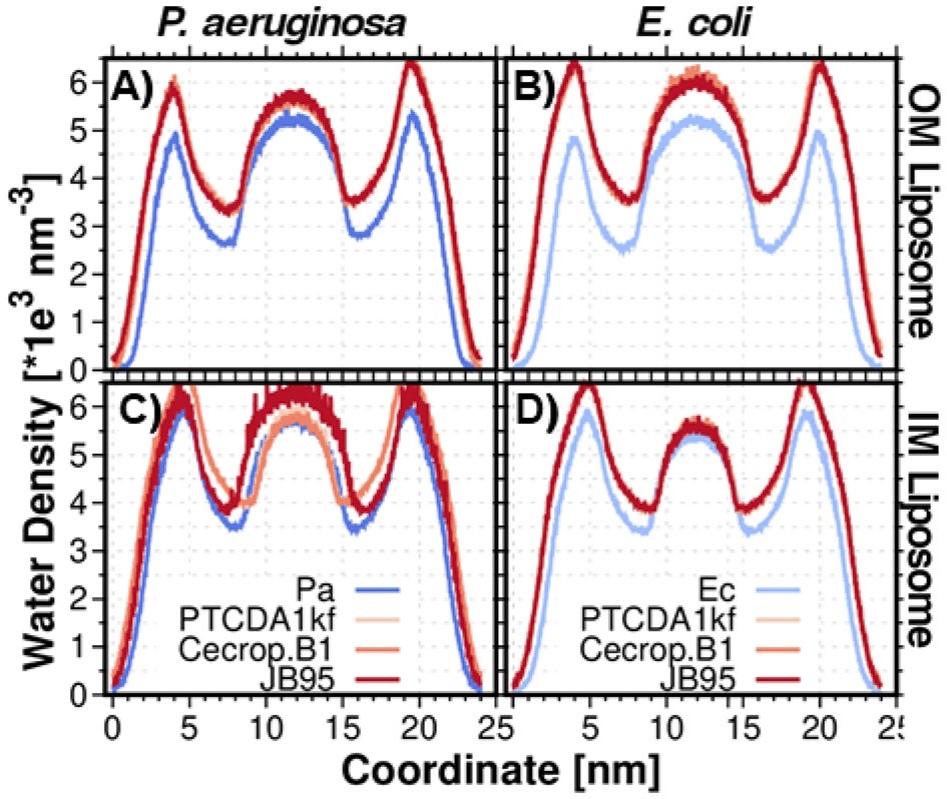
Water Density. (**A**–**B**) *P. aeruginosa* OM and IM liposomes, respectively. (**C**–**D**) *E. coli* OM and IM liposomes respectively. Analysis performed on the last μs of the MD production.

AMPs can interact with the bacterial membrane through different mechanisms, resulting in membrane disruption and enhanced membrane permeability^1^. The membrane disruption leads to the formation of a large number of cavities. As a result, water translocation across the bilayer takes place, and the membrane becomes leaky, resulting in membrane dysfunction^37,38^. Hence, we studied the effect of the AMPs on water permeation in the OM and IM liposomes. The water density profiles are represented in Fig. 9. For all models, the water number density is higher than in the AMP-free liposome, in agreement with the membrane disruption and water translocation observations. Regarding OM liposomes, membranes surface hydration and water intake show a direct correlation with the AMPs deep of interfacial adsorption and thus, with the Δ(APL_*AMP.free* _− APL _*w.AMP*_). As mentioned before, *E. coli* liposomes are more susceptible to AMP penetration that *P. aeruginosa* liposomes, inherent to their lower cardiolipin content (5 and 11% in *E. coli* and *P. aeruginosa*, respectively, Table S1, S2) and the acylated degree of the lipid A molecules (hexa-acylated in the case of *E. coli* and tetra-acylated in *P. aeruginosa*, Figure S2). Consequently, *E. coli* is more permeable to water than *P. aeruginosa*, in the AMPs presence. As for IM liposomes, AMPs interaction does not seem to affect the water density in the inner cavity and, therefore, neither in the water intake, except for JB95 in the IM *P. aeruginosa*. The results could be attributed to the lipid ordering due to the *cis–trans* conversion of unsaturated phospholipids after the incorporation of the AMPs and confirmed with P_2_ calculations. The conversion mechanism appears to have greater relevance in the IM than in the OM liposomes, according to the higher observed Δ(APL_*AMP.free* _− APL _*w.AMP*_*,* Fig. 7A,B) values. Nevertheless, this effect may be the result of a major content of unsaturated phospholipids in IM liposomes than in OM liposomes. Conversely, the water intake and permeability seem to increase in the *P. aeruginosa* IM, in the presence of JB95. As described before, the peptide remains on the membrane surface and generates the lipid package disorder, which involves an increase of APL value, and therefore an increment of water permeability. We also calculated the radial Ca^2+^ number density in the presence of the AMPs, to study the AMPs effect on the cation-membrane interaction (Figure S5). The pronounced curvature of the Ca^2+^ density profiles correlates with the AMPs penetration (See Supporting information, Figure S5).

## Conclusions

Coarse-grained (CG) molecular dynamics (MD) simulations have enabled us to characterize important features of the physico-chemical properties of ESKAPE Gram-negative bacterial membranes. We have described bacterial membrane infective vesicles by using liposome models. We built both OM and IM liposomes mimicking the lipid composition reported experimentally for strains of *E. coli, K. pneumoniae*, *A. baumannii*, and *P. aeruginosa*. This representative set of ESKAPE Gram-negative bacteria have lipid A that differ in the acylation pattern and length of acyl chains. Furthermore, the effect of cholesterol incorporation on liposomal morphology and dynamic properties was also explored. Based on these computational models, and on reported experimental data, we have discovered that both the lipid A structure, and the cardiolipin content and cholesterol acquisition from hosts in bacterial liposomes are the main factors determining the AMP resistance. The small-sized liposomes, such as IM liposomes, were found to exhibit higher diffusion than OM liposomes, whereas bacterial liposomes with a given cholesterol content show a high diffusion coefficient. Additionally, the reported antimicrobial peptides Cecropin B1, JB95, and PTCDA1-kf, have been used to unveil their effect on membrane disruption. We have also revealed that ESKAPE bacteria show increased lipid ordering in the presence of AMP peptides, as deduced by increased P2 values and decreased APL, compared with liposomes in the absence of AMPs. Changes in membrane order could be a response to maintain membrane organization and structural integrity of the vesicles as part of a bacterial resistance mechanism.

To the best of our knowledge, this systematic study is pioneering in proposing computational liposome models accounting for molecular and physico-chemical determinants of the bacterial membrane and its role in AMR in ESKAPE Gram-negative bacteria. We therefore hope that our findings and predictions will motivate and promote further experimental and theoretical studies addressing lipid A-dependent morphology and its effect on bacterial diffusion and AMP resistance for the design and discovery of new AMPs.

## Methods

### Lipid A AA-CG mapping

LPS modeler module from CHARM-GUI^39^ was used to obtained AA models of the five lipid A species studied in this work. AA models are described by the CHARM36 force field^40^ and partial charges on each atom were calculated using first principles Hartree–Fock (HF) and a basis set of 6–31 + g(d) as implemented in Gaussian package^41^. The lipid A molecule is placed in a cubic box and solvated with a TIP3P water molecules and a 0.15 M of NaCl was added together with the counter ions to neutralize the lipid A charges. The AMBER16^42^ package and a *npT* (constant number of particles, pressure, and temperature) ensemble was used to accumulate 200 ns of MD production.

The equilibrated AA lipid A molecule was coarse-grained using the PyCGtool^43^ and the MARTINI many-to-one mapping protocol^44^. The CG lipid A is placed in a cubic box and solvated with normal MARTINI water (W) and neutralized with the corresponding counter-ions and with a salt concentration of 0.15 M NaCl. Then, the GROMACS molecular dynamics package (version 4.5 and 5.x)^45^ was used to perform all the CG simulations. Energy minimization was performed using the steepest-decent algorithm with a 20 fs time-step until the maximum force on any bead was below the tolerance parameter of 10 kJ mol^−1^ nm^−1^. Periodic boundary conditions were applied in all three dimensions. The systems were maintained at 1 bar using the Berendsen barostat with time constant, *τ*_p_ = 3.0 ps. Temperature was maintained at 310 K by independently coupling the lipid and the solvent to an external velocity rescaling thermostat with *τ*_T_ = 1.0 ps. The neighbor list was updated every 10 steps using 1.4 and 1.2 nm for short-range van der Waals and electrostatic cutoffs, respectively. The production *npT* simulations were performed for 1 μs for all the systems.

### Liposome construction

A routine in Packmol^46^ was used to build a starting bacterial liposome with 400 molecules in each side of the liposome membrane, outer and inner, with a total of 800 molecules (Figure S6). The systems were subsequently neutralized by adding counter-ions, Ca^2+^ and Cl^−^, and solvated with CG water particles. The initial liposomes radii were approximately 12 nm. Liposome lipid compositions for the studied Gram-negative bacteria in ESKAPE were taken from the literature^14,15^ (summarized in Tables S1–4). OM and IM liposomes contained the main glycerophospholipids, phosphatidylethanolamine (PE), phosphatidylglycerol (PG), and phosphatidylcholine (PC) in the *P. aeruginosa* case. To mimic OM liposomes, the outer leaflet contained a lipid A-to-lipid ratio of 3:1 and, IM liposomes contained their corresponding mix of lipid-based composition. Cholesterol was inserted with a lipid-to-cholesterol ratio of 2:1 in inner leaflet.

### AMP-liposome set-up

Peptides PTDCA1-kf^31^, Cecropin B1^28^, and polymyxin JB95^27^, were used as AMPs for *E. coli*, *P. aeruginosa* and *K. pneumoneae*. They were submitted to AAMD simulations in water, in order to reach stable secondary structure. AA AMPs models were built using AmberTools software package^42^. Both Xleap and tleap were used to build the initial 3D structures. The Amber force field ff14SB^47^ was used to represent the amino acids. A TIP3P model was used to represent water molecules. The simulations included 3 phases: minimization, heating and production. The phase of minimization included 2000 maximum minimization cycles with a cutoff distance of 10 Å (common cutoff in all simulation phases). The AAMD simulations were run using the pmemd.cuda code as implemented in AMBER16^42^, running on single Nvidia® GPUs. The heating used an NVT ensemble, for keeping volume and temperature value constant for 20 ps from 0 to 310 K. The production included an npT ensemble, with constant pressure and temperature, at 1 atm and 310 K until obtaining a stable secondary structure (Figure S4). All simulations had a 2 fs timestep integrator and long-range Coulomb contributions were processed through PME^48^. All systems were neutralized by adding counter-ions, Cl^−^.

The script martinize.py^44^ was used to mapping the all-atom coordinates to CG models. Then, four AMP molecules were randomly placed around the liposomes, using the gmx *insert-molecules* tool as implemented in GROMACS 5.x^45^. Liposomes were preequilibrated during 5 µs before peptide addition, and the proportion of Ca^2+^ and Cl^-^ neutralizing ions was maintained. After the AMPs incorporation, the systems were subsequently neutralized by adding Cl^-^ ions, to counterbalance the cationic peptides charge, and solvated with normal MARTINI water^44^.

### Simulation set-up

All CG molecular dynamics (MD) simulations were performed using the GROMACS simulation package (version 4.5 or 5.x)^45^. All CG models for liposome components were represented using MARTINI force field^44^. CG topologies and parameters for all phospholipids species, with the exception of lipid A molecules, were obtained from www.cgmartini.nl. Lipid A parametrization was performed following the protocol reported in^19^, where MARTINI CG parameters of eight different bacterial species were successfully generated and validated by comparison with all-atom MD simulations results.

An energy minimization using the steepest descent algorithm over 1000 steps was carried out for the initial structure. Then, an NPT equilibration without position restrains was run for 50 ns. The particle mesh Ewald (PME) method^48^ was used to calculate long-range electrostatic interactions, using a maximum grid spacing of 2.5 Å, fourth-order (cubic) interpolation for the fast Fourier transforms and a relative dielectric constant of 15. A dielectric constant of 15 is used for explicit screening to balance the increased hydration strength of many of the CG particle types. The temperature was kept constant at 323 K by coupling the phospholipids, lipid A, ions and the solvent independently to an external bath using the Berendsen algorithm with a coupling constant of 1 ps. Isotropic scaling was used for the pressure (1 bar) with a coupling constant of 2.0 ps and a compressibility of 3e^−4^ bar following the Berendsen algorithm. The temperature range used in the original Martini force field parameterization for lipids was 270–330 K^45^. The calculated transition temperature for most of liposomes lipids used in this study is around the same range. Therefore, the 323 K temperature selected for our simulations could be considered physiologically admissible. The dynamics were integrated using the velocity Verlet integrator, with a time step of 20 fs and bonds constrained using the LINCS algorithm. Thus, production dynamics were performed at constant pressure and temperature (NPT ensemble). Independent trajectories were generated for each studied model using different seed numbers for the initial velocity assignment. The Root-mean square deviation (RMSD) of the liposomes was stablished as a parameter to determine the equilibrated state of all systems (See Supplementary Information, Figure S9, where the systems reached an equilibrated state). Minimum distances between our main liposome and its periodic images are included in Supplementary information (Figure S10). As can be observed, all systems were isolated during the simulation time, with minimum distances greater than the cutoff distance 10 Å.

### Analysis

Post simulation analyses were performed using in-built GROMACS^45^ utilities and in-house python/bash scripts. Molecular visualization and graphics were generated using visual molecular dynamics (VMD) software^49^. All the post simulation analysis was performed on the last μs of the MD production.

#### Secondary structure analysis

A Database of Secondary Structure of Proteins (DSSP)^50,51^ program as implemented in AMBER16^42^ was used to calculate peptides secondary structures along AAMD simulations. DSSP is a public database of secondary structure assignments for all protein entries in the Protein Data Bank accessible at DSSP (umcn.nl).

#### Area per lipid (APL)

 Solvent-accessible surface area (SASA) was measured by rolling a probe sphere with the radius of the CG water molecule (0.45 nm) around the liposome molecules. The tool gmx *sasa* as implemented in GROMACS was used. The calculated areas were divided by the number of lipids present in the liposome to obtain an estimated APL.

#### Density

Nnumber radial density was calculated with the gmx densmap tool as implemented in GROMACS. It computes 2D number-density maps as an axial-radial density map.

#### Membrane thickness

Membrane thickness was estimated from number radial density profiles as represented in Figure S7. Maximum and minimum values from the last µs of MD production were predicted and averaged using an in-house python program.

#### Diffusion constant

The lateral diffusion coefficient in the liposome membrane was calculated from the mean square displacement on a spherical surface in which the lateral diffusion coefficient D is given by, $D = \left\langle {\frac{{4R^{2} \theta^{2} }}{t}} \right\rangle$, where *θ* is the angle between the membrane normal vector at *t* = 0 and *t* =* t*, defined as the connecting vector between the center-of mass of the liposome and the center-of-mass of the lipid, and R is the radius of the vesicle. The diffusion was fitted from the mean square displacement in the range of 200–800 ns. It was calculated on each liposome component and thus, the mean lateral diffusion coefficient is reported, 〈*D*〉.

#### AMP interfacial deep of adsorption

Iit is calculated by subtracting to the liposome radius the distance among the center-of-mass (com) of each AMP to the com of the liposome in each frame, thus for each AMP, AMP-Adsorption = *R*_Liposome_ − *r*_AMP_COM-Liposome_COM_. Then, the data for each AMP is collected and represented by histograms.

#### Order parameter

The second-rank order parameter, $P_{2} = \frac{1}{2}\left\langle {3\cos^{2} \theta - 1} \right\rangle$, was computed for consecutive bonds with *θ* being the angle between the direction of the bond and the vector connecting the center of the bond with the center of the liposome.

## Supplementary Information

Supplementary Information.

## Data Availability

Data used to support this study are included in the article.

## Abbreviations

AA: All-atom
Ab: *A. baumannii*
AMP: Antimicrobial peptide
AMR: AntiMicrobial Resistance
APL: Area per lipid
CDL: Cardiolipin
CG: Coase-grain
CHOL: Cholesterol
Com: Center-of-mass
< D >: Lateral diffusion coefficient
DNA: Deoxyribonucleic acid
DOPC: 1,2-Dioleoyl-sn-glycero-3-phosphocholine
DOPE: 1,2-Dioleoyl-sn-glycero-3-phosphoethanolamine
DOPG: 1,2-Dioleoyl-sn-glycero-3-phosphoglycerol
DPPC: 1,2-Dipalmitoyl-sn-glycero-3-phosphocholine
DPPE: 1,2-Dipalmitoyl-sn-glycero-3-phosphoethanolamine
DPPG: 1,2-Dipalmitoyl-sn-glycero-3-phosphoglycerol
DSSP: Database of Secondary Structure of Proteins
Ec: *E. coli*
ESKAPE: *Enterococcus faecium, Staphylococcus aureus, Klebsiella pneumoniae, Acinetobacter baumannii, Pseudomonas aeruginosa,* and *Enterobacter spp*
IM: Inner membrane
Kp: *K. pneumoniae*
L: Thickness
LC: Lethal concentration
Lchain: Length of chain
LPS: Lipopolysaccharide
MD: Molecular dynamics
MIC: Minimum inhibitory concentration
MSD: Radial mean square displacement
Mv: Membrane vesicle
Nchain: Number of chains
OM: Outer membrane
P2: Order parameter
Pa: *P. aeruginosa*
PC: Phosphatidylcholine
PE: Phosphatidylethanolamine
PG: Phosphatidylglycerol
PME: Particle mesh Ewald
POPE: 1-Palmitoyl-2-oleoyl-sn-glycero-3-phosphatidylethanolamine
POPG: 1-Palmitoyl-2-oleoyl-sn-glycero-3-phosphatidylglycerol
RMSD: Root-mean square deviation
SASA: Solvent-accessible surface area
WHO: World Health Organization
