# Supplementary material for: Lipid-A-dependent and cholesterol-dependent dynamics properties of liposomes from gram-negative bacteria in ESKAPE
Source: Sci Rep. 2022 Nov 14;12:19474. doi: 10.1038/s41598-022-22886-7 (PMC9663605; doi:10.1038/s41598-022-22886-7)
Supplement: Supplementary file 1 — Supplementary Information. [file 41598_2022_22886_MOESM1_ESM.pdf]

Supplementary Information

Lipid-A-Dependent and Cholesterol-Dependent Dynamics Properties of Liposomes from Gram-Negative Bacteria in ESKAPE

Juan Felipe Franco-Gonzalez,* Alejandra Matamoros-Recio, Angel Torres-Mozas, Blanca Rodrigo-Lacave, Sonsoles Martin-Santamaria*

Department of Structural and Chemical Biology, Centro de Investigaciones Biológicas Margarita Salas, CIB-CSIC. C/ Ramiro de Maeztu, 9. 28040-Madrid, Spain.

E-mail: j.f.francogonzalez@gmail.com; smsantamaria@cib.csic.es

Table of contents.

S1. General chemical structure of Gram-negative bacterial lipopolysaccharides.
S2. Atomistic and Coarse-Grained representation of lipid A structures.
S3. Chemical structure of the Antimicrobial peptides studied in this work.
S4. Tertiary structure of the Antimicrobial peptides, after MD simulations in water.
S5. Radial Ca^{2+} density profiles in the presence of the Antimicrobial peptides.
S6. Coarse-grained liposome model of the outer membrane of *A. baumannii*, with cholesterol.
S7. Membrane thickness estimation (L) from density profiles.
S8. Lipids and cholesterol domains formation in liposomes models.
S9. RMSD values of the liposomes along MD simulations.
S10. Minimun distance between the liposomes and their periodic images.

Tables S1-4. Lipid composition of the liposomes models, mimicking the bacterial inner and outer membranes.
Table S5. Liposomes membrane thickness estimation.
Table S6. Amino acid sequences and physical characteristics of the investigated AMPs.
Table S7. Experimental MIC Values and Lethal Concentrations of the AMPs, as reported in bibliography.

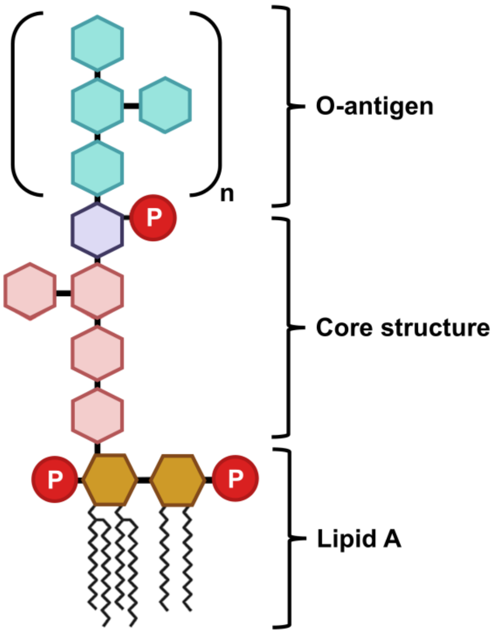

Figure S1. Schematic representation of the general chemical structure of LPS. (Smooth)-type LPSs are built up of three distinct moieties, termed lipid A, core, and the O-antigen. In cases of absent or truncated O-chains, the terminology employed is (rough)-type LPS.

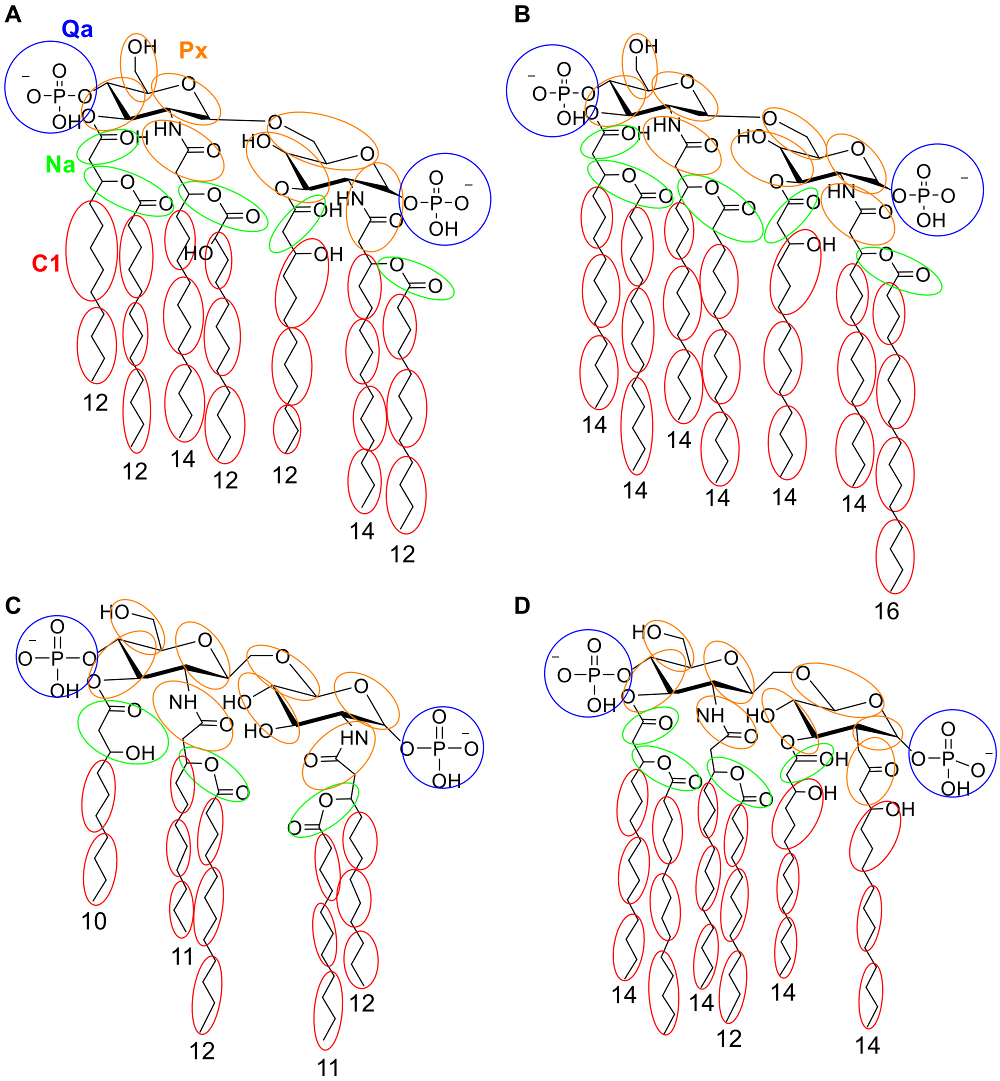

Figure S2. Atomistic representation of Lipids A of different bacterial species and their corresponding CG mapping scheme. **(A)** Lipid A of *A. baumannii*, **(B)** Lipid A of *K. pneumoniae*, **(C)** Lipid A of *P. aeruginosa*, **(D)** Lipid A of *E. coli*.

A) Cecropin-B1

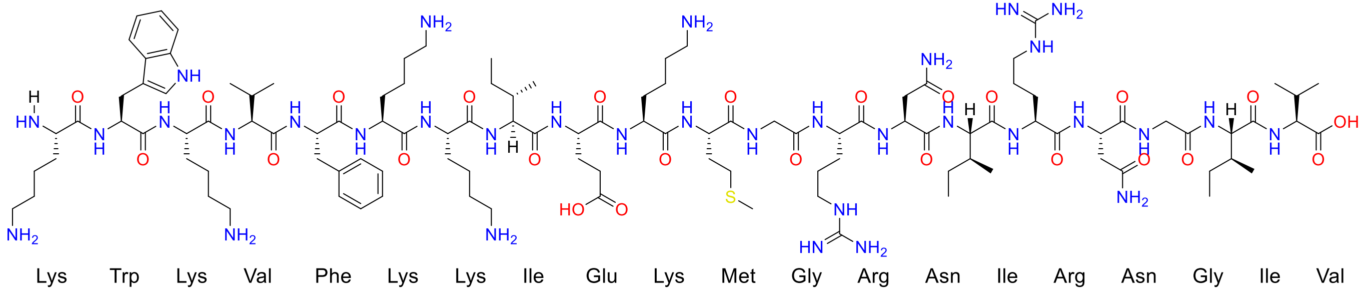

B) JB-95

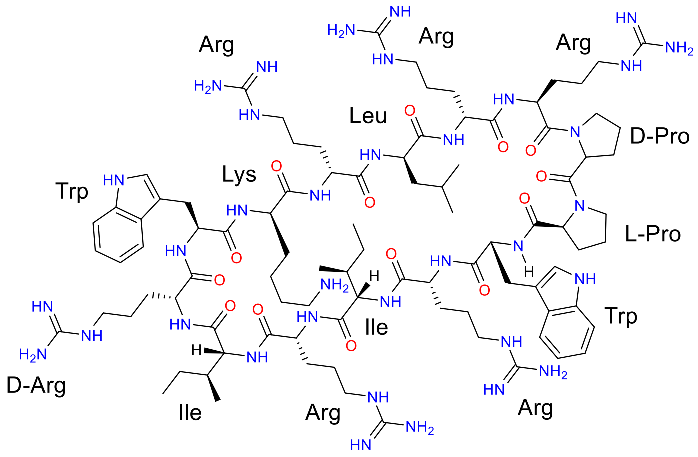

C) PTCDA1-kf

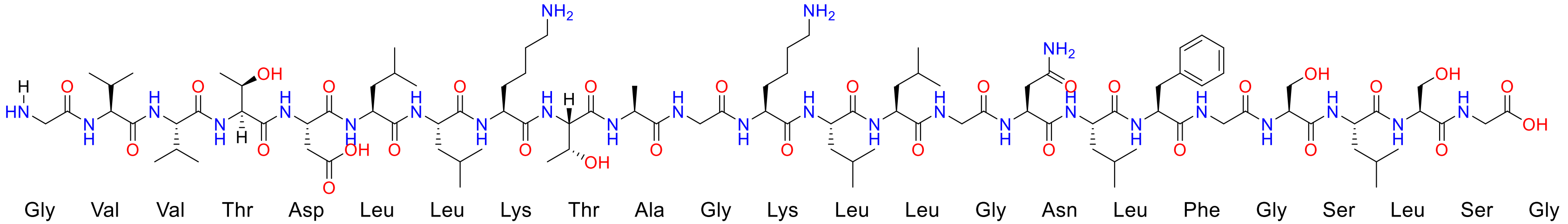

Figure S3. Structure of the AMPs selected for this work. Structure of **(A)** Cecropin-B1, **(B)** JB-95, and **(C)** PTCDA1-kf.

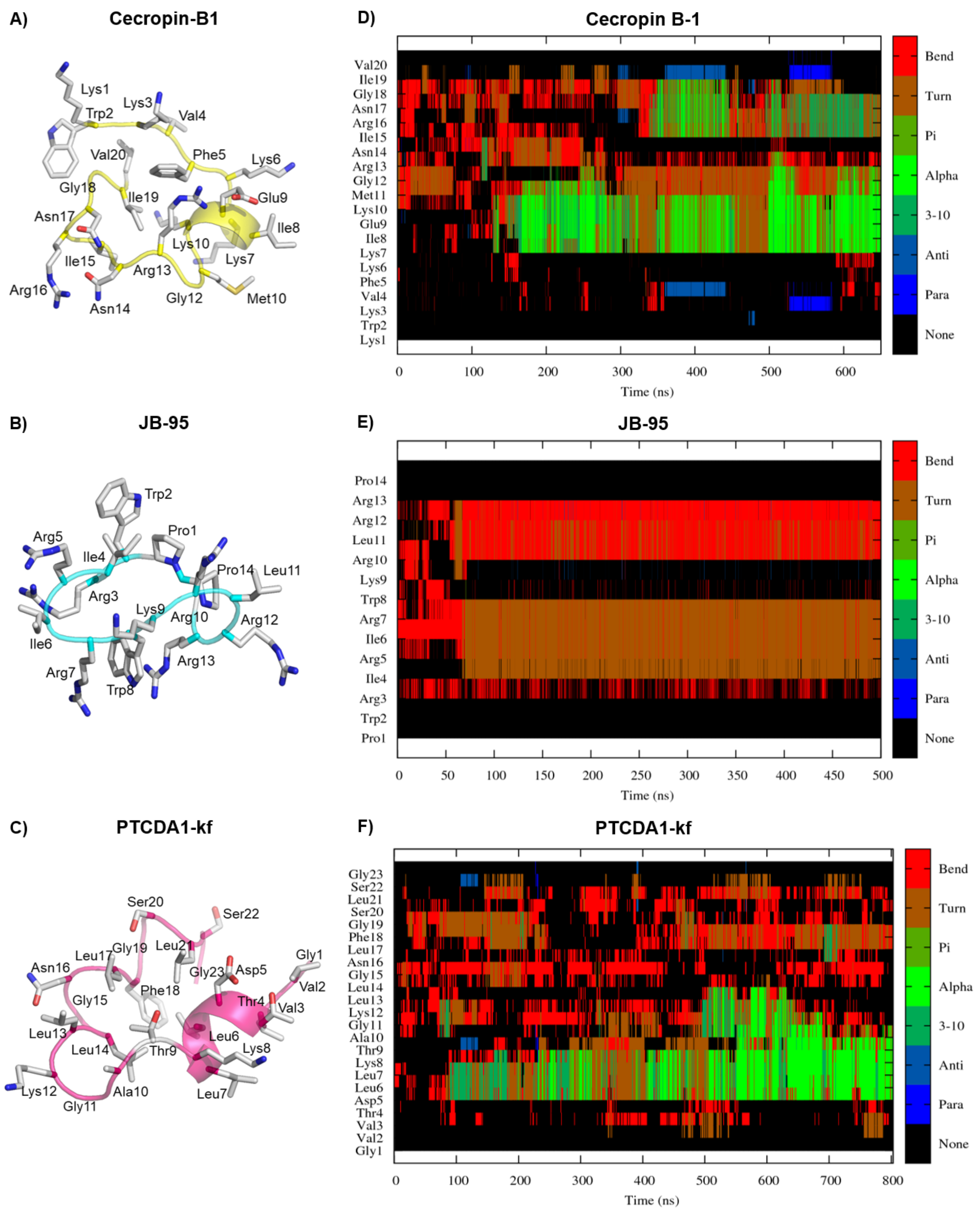

Figure S4. All-atom MD simulations of the AMPs. Final structures after **(A)** 650 ns for Cecropin-B1, **(B)** 500 ns for JB-95, and **(C)** 800 ns for PTCDA1kf **(D-F)** Secondary structure prediction (DSSP) analysis over simulations.

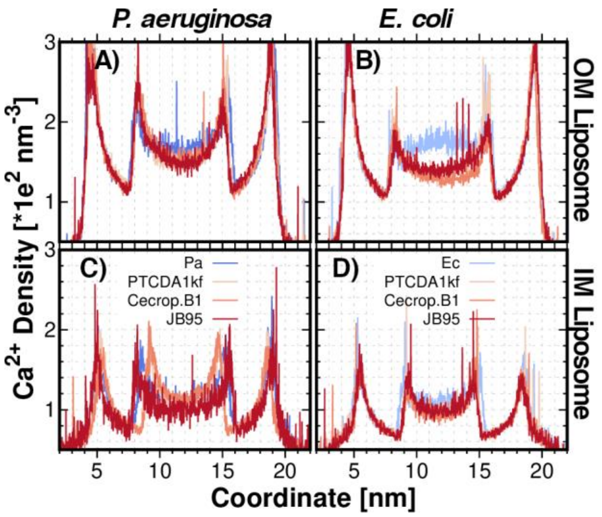

Figure S5. Radial Ca^{2+} number density in the presence of the AMPs. A-B) *P. aeruginosa* OM and IM liposomes, respectively. C-D) *E. coli* OM and IM liposomes respectively. In the hydrophobic core region of OM liposomes, a pronounced curvature for the Ca^{2+} profile is observed when AMPs interacts with the liposome membrane, pointing to a reduction in the Ca^{2+} density in the inner cavity. The curvature pronunciation seems to correlate with the AMPs penetration. The deeper penetration, the closer to the liposome inner cavity. As result, the AMPs could displace the Ca^{2+} cations from the inner cavity, due to the electrostatic repulsion. Moreover, some cationic AMPs are known to permeabilize the bacterial outer membrane via a competitive displacement of divalent cations.[33] The positively charged residues in the peptide bind the negatively charged LPS head groups leading to a divalent counterions displacement, along with structural changes in membrane integrity.[1] Therefore, the Ca^{2+} displacement, together with the electrostatic repulsion between the cationic peptides and the Ca^{2+} ions could decrease the Ca^{2+} density in the liposomes inner cavity. As for IM liposomes, the same effect is observed in the *E. coli* liposomes, but with a less pronounced curvature for the Ca^{2+} density in the inner cavity. Conversely, not variations in Ca^{2+} density values are observed for the *P. aeruginosa* IM, which may indicate that the AMPs does not penetrate the membrane enough to repel and displace the divalent cations from the inner cavity.

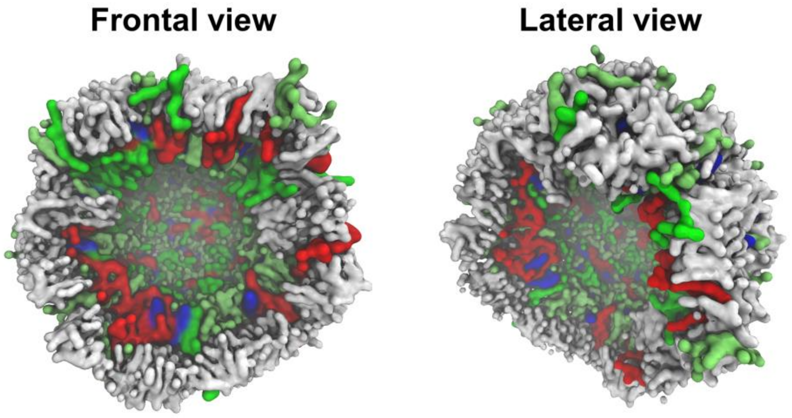

Figure S6. Snapshot of a coarse-grained liposome model used in this work. Cross sectional view of the liposome. The lipid composition corresponds to the outer membrane of *A. baumannii*, with cholesterol. Lipid A is in grey, POPE and POPG in green, cholesterol in red and cardiolipin in blue.

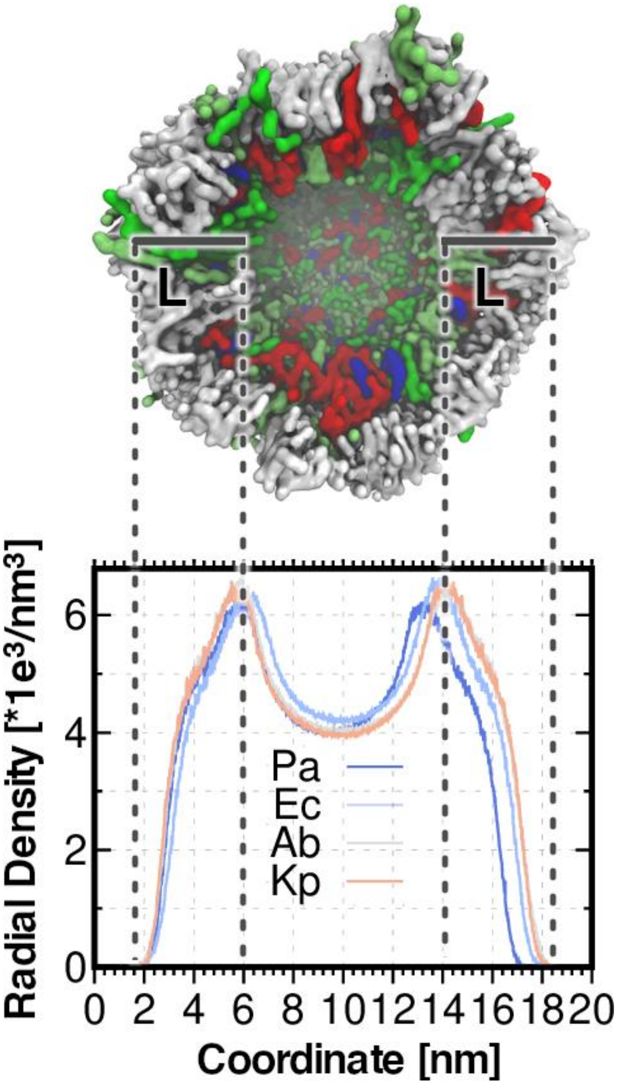

Figure S7. Illustrative scheme for the membrane thickness estimation (L) from density profiles. *Top*: cross sectional view of the liposome. *Bottom*: liposome components density (same as Figure 2C). Analysis performed on the last µs of the MD production.

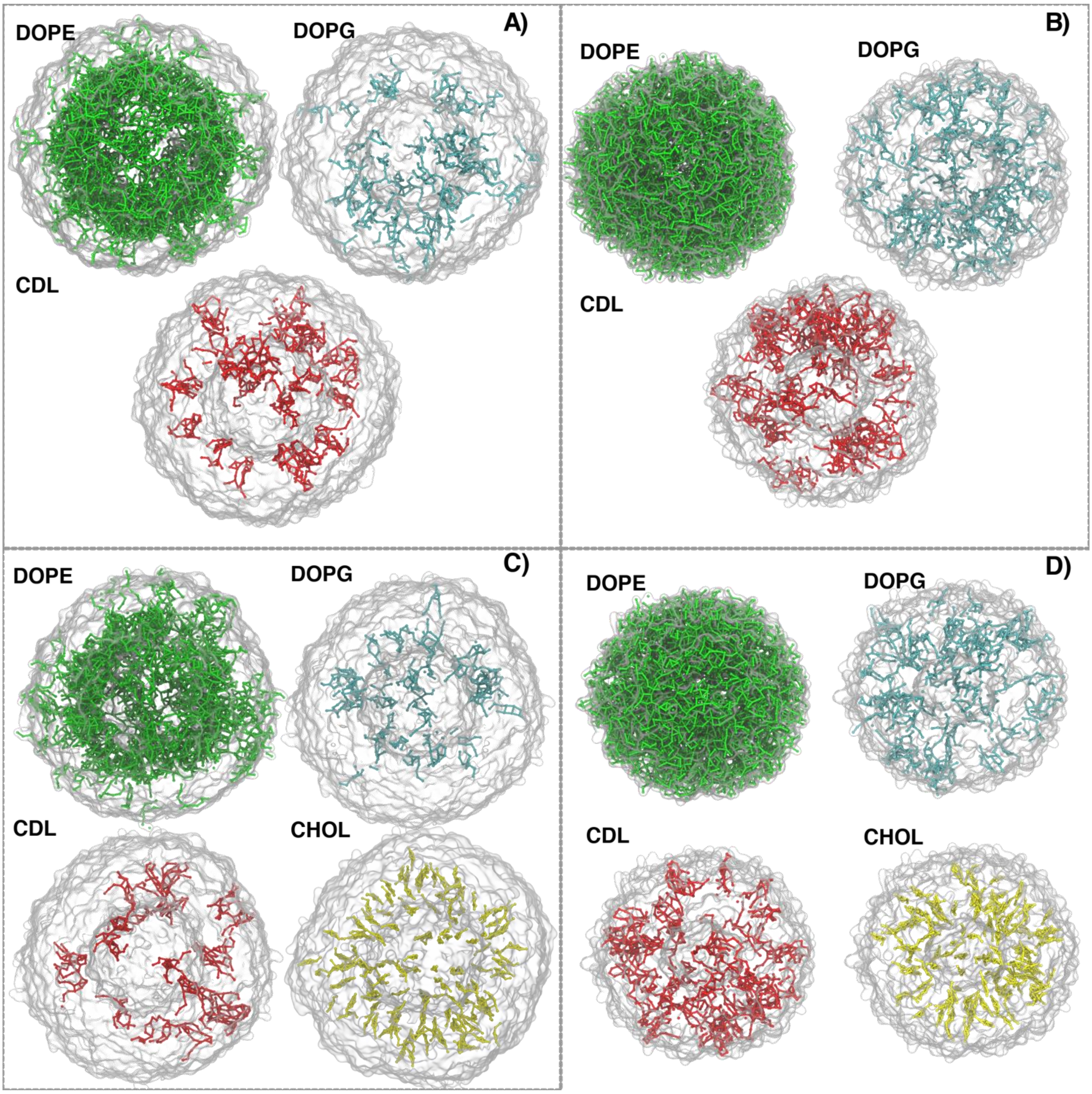

Figure S8. Lipids and cholesterol domains formation exemplified by *K. pneumoniae* liposomes models. Cholesterol-free OM (A) and IM (B) liposomes respectively. With cholesterol (C) and (D) respectively. External view. Cholesterol in yellow, DOPE in green, DOPG in cyan and CDL in red. Water and Ca^{+2} beads are not included for clarification. 3D structures from the last equilibrated frame.

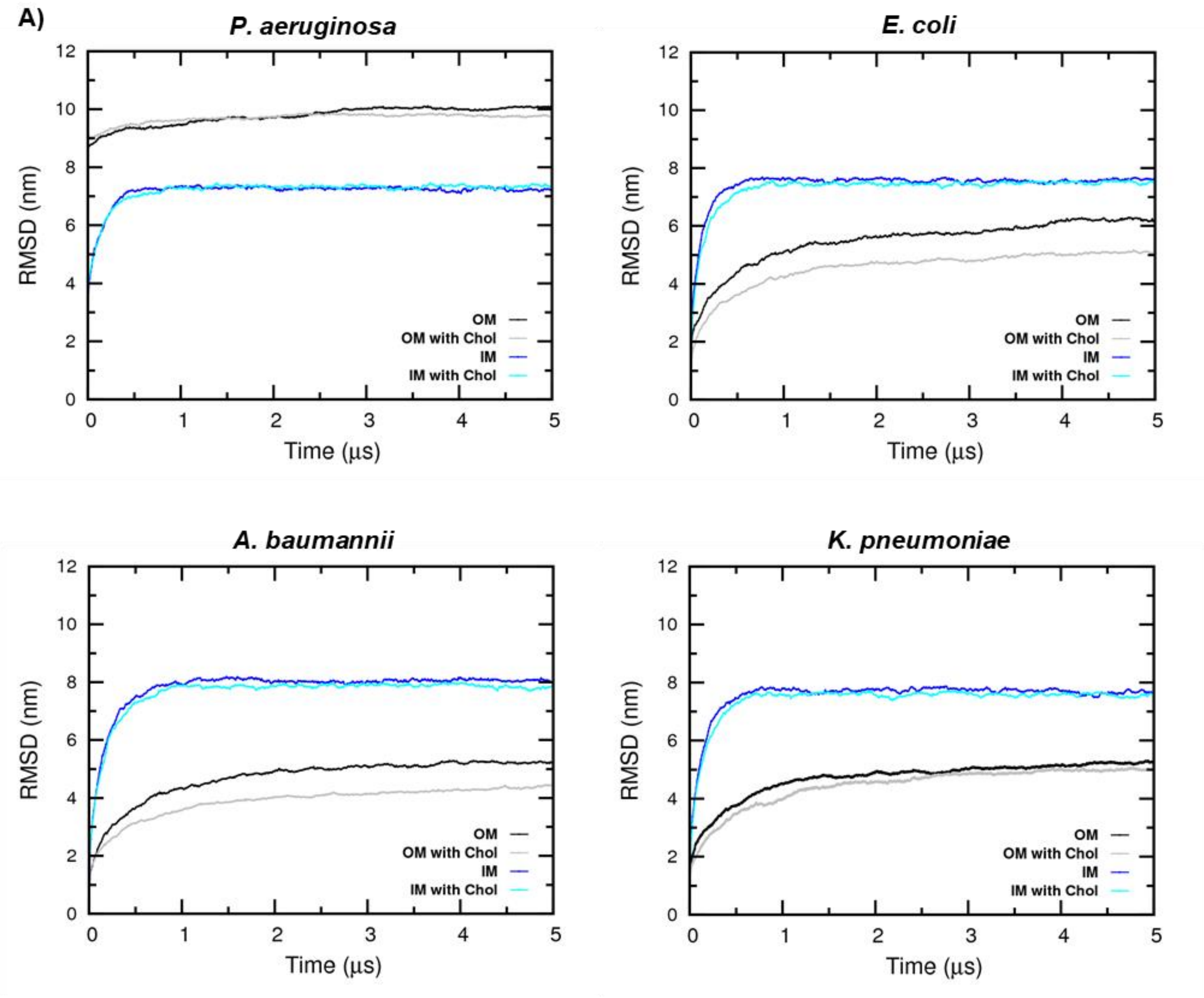
A)
P. aeruginosa
E. coli
A. baumannii
K. pneumoniae
RMSD (nm)
Time (μs)
OM
OM with Chol
IM
IM with Chol

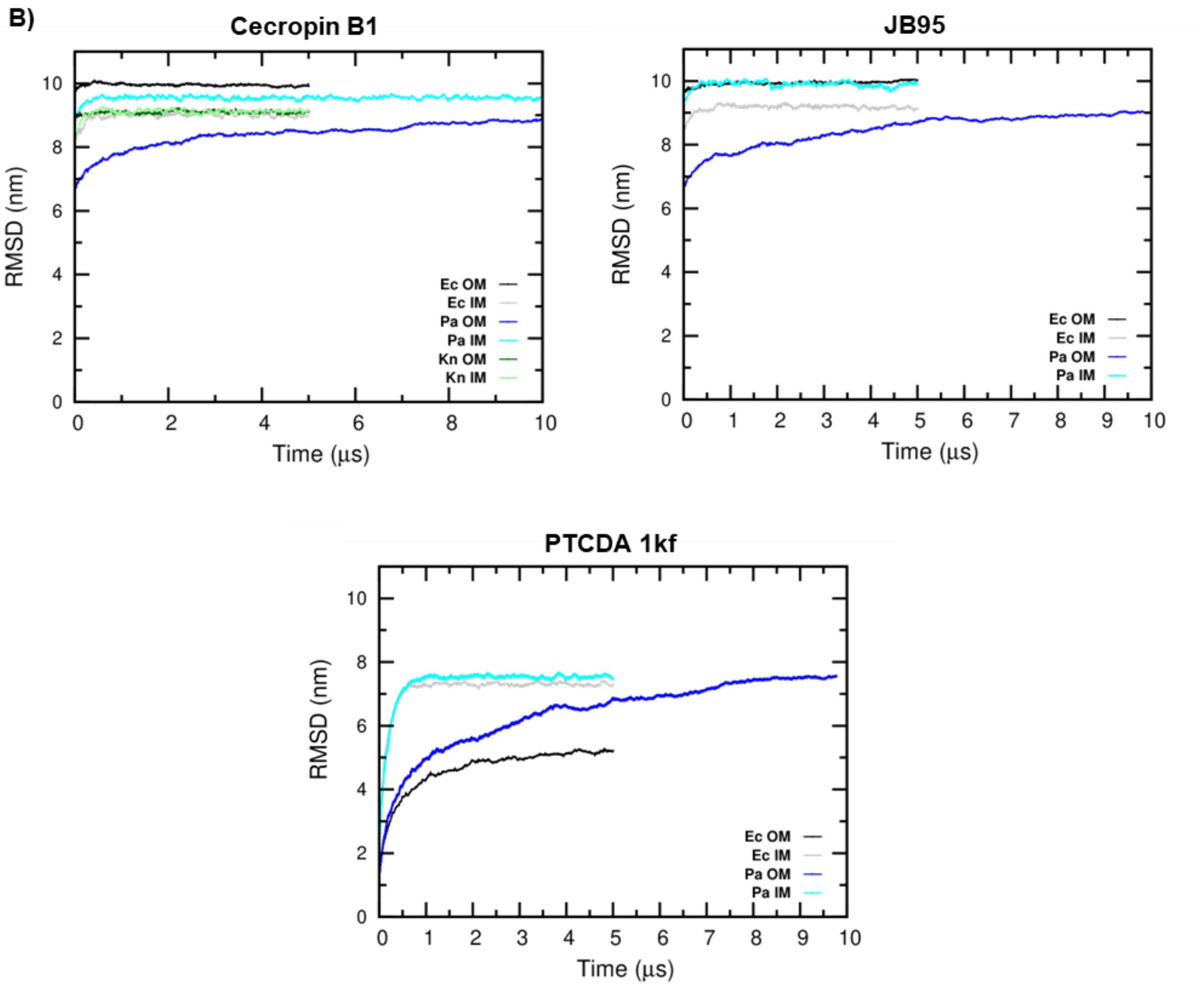

Figure S9. Stability of liposomes along MD simulations. RMSD of the liposomes over simulation time, in the absence (A), and presence (B) of AMPs in *E. coli* (Ec), *P. aeruginosa* (Pa), and *K. pneumoniae* (Kp). All the systems reached an equilibrated state.

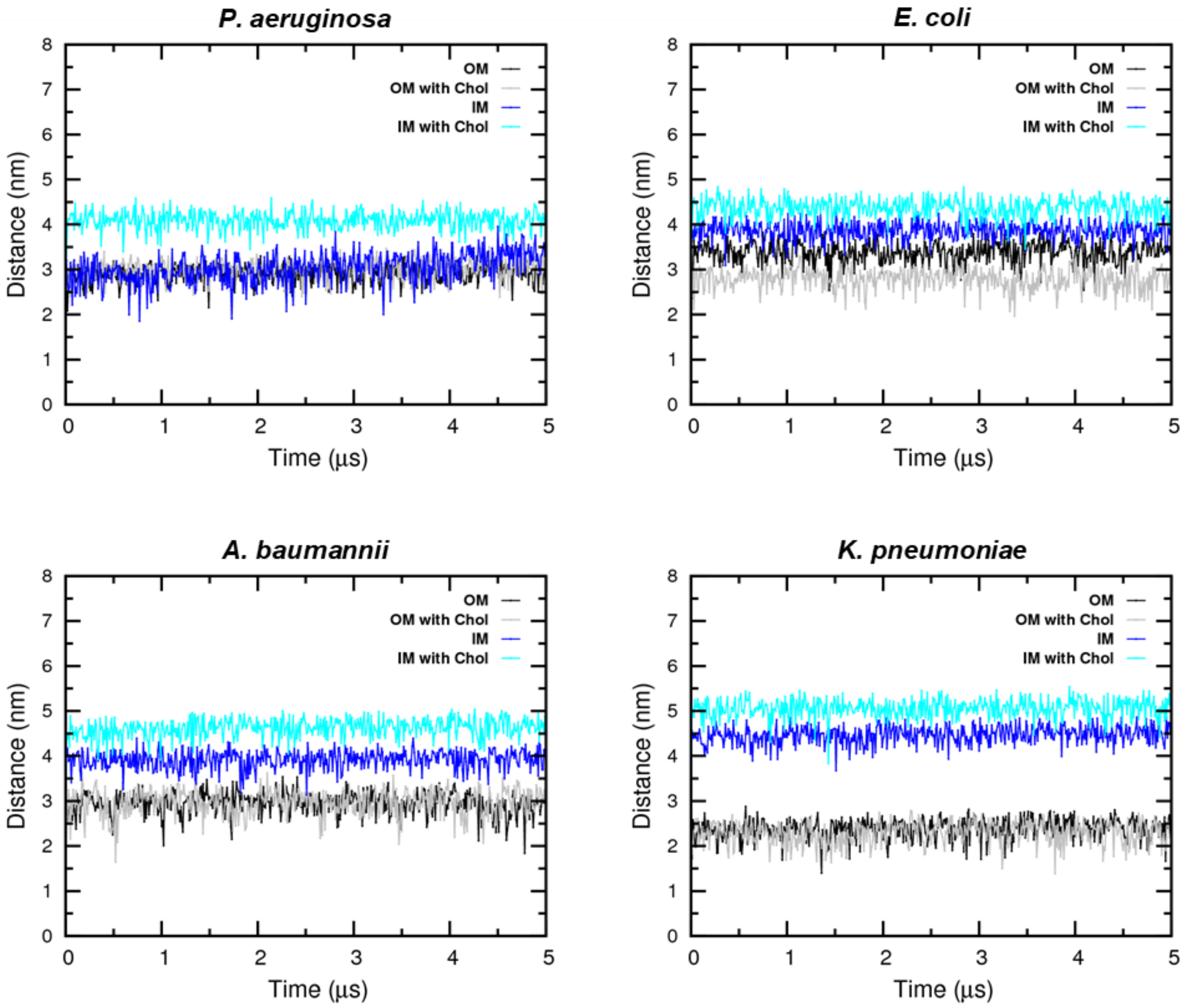

Figure S10. Monitorization of liposomes during MD simulations. Minimun distance between the liposomes and their periodic images. The systems were isolated during the simulation time, with minimum distances greater than the cutoff distance 10 Å.

Tables S1-4. Lipid composition of the inner membrane and outer membrane liposome models.

Table S1. *P. aeruginosa*

**Liposome**	**Leaflet**	**Proportion (%)**	**Number molecules**
IM	Inner	DPPE/DOPE/DPPG/DOPG/DPPC/DOPC/CDL: 29/24.25/13/11/6.5/5.25/11	116/97/52/44/26/21/44
	Outer	DPPE/DOPE/DPPG/DOPG/DPPC/DOPC/CDL :29/24.25/13/11/6.5/5.25/11	116/97/52/44/26/21/44
IM+CHOL	Inner	DPPE/DOPE/DPPG/DOPG/DPPC/DOPC/CDL/CHOL: 19.25/16.25/8.75/7.25/4.25/3.5/7.25/33.5	77/65/35/29/17/14/29/134
	Outer	DPPE/DOPE/DPPG/DOPG/DPPC/DOPC/CDL: 29/24.25/13/11/6.5/5.25/11	116/97/52/44/26/21/44
OM	Inner	DPPE/DOPE/DPPG/DOPG/DPPC/DOPC/CDL: 29/24.25/13/11/6.5/5.25/11	116/97/52/44/26/21/44
	Outer	DPPE/DOPE/DPPG/DOPG/DPPC/DOPC/CDL/LPA: 7.25/6/3.25/2.75/1.75/1.25/2.75/75	29/24/13/11/7/5/11/300
OM+CHOL	Inner	DPPE/DOPE/DPPG/DOPG/DPPC/DOPC/CDL/CHOL: 19.25/16.25/8.75/7.25/4.25/3.5/7.25/33.5	77/65/35/29/17/14/29/134
	Outer	DPPE/DOPE/DPPG/DOPG/DPPC/DOPC/CDL/LPA: 7.25/6/3.25/2.75/1.75/1.25/2.75/75	29/24/13/11/7/5/11/300

Table S2. *E. coli*

**Liposome**	**Leaflet**	**Proportion (%)**	**Number Molecules**
IM	Inner	POPG/POPE/CDL:15/80/5	60/320/20
	Outer	POPG/POPE/CDL:15/80/5	60/320/20
IM+CHOL	Inner	POPG/POPE/CDL/CHOL: 10.05/53.6/3.35/33	40/214/14/132
	Outer	POPG/POPE/CDL:15/80/5	60/320/20
OM	Inner	POPG/POPE/CDL:15/80/5	60/320/20
	Outer	POPG/POPE/CDL/LPA:3.75/20/1.25/75	15/80/5/300
OM+CHOL	Inner	POPG/POPE/CDL/CHOL:10.05/53.6/3.35/33	40/214/14/132
	Outer	POPG/POPE/CDL/LPA: 3.75/20.0/1.25/75	15/80/5/300

Table S3. *K. pneumoniae*

**Liposome**	**Leaflet**	**Proportion (%)**	**Number molecules**
IM	Inner	DOPE/DOPG/CDL:82/12/6	328/48/24
	Outer	DOPE/DOPG/CDL1:82/12/6	328/48/24
IM+CHOL	Inner	DOPE/DOPG/CDL/CHOL: 54.5/8/4/33.33	218/32/16/134
	Outer	DOPE/DOPG/CDL:82/12/6	328/48/24
OM	Inner	DOPE/DOPG/CDL: 82/12/6	328/48/24
	Outer	DOPE/DOPG/CDL/LPA:20.5/3/1.5/75	82/12/6/300
OM+CHOL	Inner	DOPE/DOPG/CDL/CHOL:54.5/8/4/33.33	218/32/16/134
	Outer	DOPE/DOPG/CDL/LPA:20.5/3/1.5/75	82/12/6/300

Table S4. *A. baumannii*

**Liposome**	**Leaflet**	**Proportion (%)**	**Number molecules**
IM	Inner	POPE/POPG/CDL:55/30/15	220/120/60
	Outer	POPE/POPG/CDL:55/30/15	220/120/60

IM+CHOL	Inner<br>Outer	POPE/POPG/CDL/CHOL:36.67/20/10/33.33<br>POPE/POPG/CDL:55/30/15	146/80/40/134<br>220/120/60
OM	Inner<br>Outer	POPE/POPG/CDL:55/30/15<br>POPE/POPG/CDL/LPA:13.75/7.5/3.75/75	220/120/60<br>55/30/15/300
OM+CHOL	Inner<br>Outer	POPE/POPG/CDL/CHOL:36.67/20/10/33.33<br>POPE/POPG/CDL/LPA:13.75/7.5/3.75/75	146/80/40/134<br>55/30/15/300

Table S5. Liposome membrane thickness estimation. Analysis performed on the last μs of production.

**Liposome**	**OM**	**OM+CHOL**	**IM**	**IM+CHOL**
P. aeruginosa	3.94 +/- 0.02	4.01 +/- 0.03	4.10 +/- 0.14	3.93 +/- 0.11
E_ coli	3.98 +/- 0.07	4.54 +/- 0.20	4.27 +/- 0.09	3.65 +/- 0.04
A. baumannii	4.06 +/- 0.05	4.43 +/- 0.06	4.00 +/- 0.24	4.01 +/- 0.30
K. pneumoniae	4.29 +/- 0.10	4.14 +/- 0.47	4.28 +/- 0.12	4.30 +/- 0.28

Table S6. Primary Sequences and Physical Characteristics of the AMPs described in this study.

	**Sequence**	**Secondary structure**[a]	**Q**[b]
**Cecropin-B1**	KWKVFKKIEKMGRNIRNGIV	CCCCCC3333TTSCTTTTCC	+6
**JB-95**	PWRIRI-R(D)-WKRLRRP-(D)	CCCCSSSCCSSSCC	+7
**PTCDA1-kf**	GVVTDLL**K**TAG**K**LLGNL**F**GSLSG	CCC13332SCTTSTTTSTCCCTC	+2

[a]SS names; "1": Helix start (H-bond donor); "2": Helix end (H-bond acceptor); "3": Ambivalent helix type (short helices); "T": Turn; "S": Bend; "C": Coil.
[b]Net charge.

Table S7. MIC Values and Lethal Concentrations reported for AMPs studied in this work.[24–27]

**AMP**	**MIC (μM)**		**LC (μM)**		
	P. aeruginosa	***E. coli***	***P. aeruginosa***	***E. coli***	***K. pneumoniae***
Cecropin B1	0.31	0.31	1.48	0.49	0.39
PTCDA1-kf	3.1	12.5	n.a	n.a	n.a.
JB95	2.0	0.126	n.a	n.a	n.a
